# Why do RuO_2_ electrodes catalyze electrochemical CO_2_ reduction to methanol rather than methane or perhaps neither of those?†

**DOI:** 10.1039/d0sc01882a

**Published:** 2020-07-30

**Authors:** Ebrahim Tayyebi, Javed Hussain, Egill Skúlason

**Affiliations:** Science Institute, University of Iceland VR-III 107 Reykjavík Iceland egillsk@hi.is; Faculty of Industrial Engineering, Mechanical Engineering and Computer Science, University of Iceland VR-III 107 Reykjavík Iceland

## Abstract

The electrochemical CO_2_ reduction reaction (CO_2_RR) on RuO_2_ and RuO_2_-based electrodes has been shown experimentally to produce high yields of methanol, formic acid and/or hydrogen while methane formation is not detected. This CO_2_RR selectivity on RuO_2_ is in stark contrast to copper metal electrodes that produce methane and hydrogen in the highest yields whereas methanol is only formed in trace amounts. Density functional theory calculations on RuO_2_(110) where only adsorption free energies of intermediate species are considered, *i.e.* solvent effects and energy barriers are not included, predict however, that the overpotential and the potential limiting step for both methanol and methane are the same. In this work, we use both *ab initio* molecular dynamics simulations at room temperature and total energy calculations to improve the model system and methodology by including both explicit solvation effects and calculations of proton–electron transfer energy barriers to elucidate the reaction mechanism towards several CO_2_RR products: methanol, methane, formic acid, CO and methanediol, as well as for the competing H_2_ evolution. We observe a significant difference in energy barriers towards methane and methanol, where a substantially larger energy barrier is calculated towards methane formation than towards methanol formation, explaining why methanol has been detected experimentally but not methane. Furthermore, the calculations show why RuO_2_ also catalyzes the CO_2_RR towards formic acid and not CO(g) and methanediol, in agreement with experimental results. However, our calculations predict RuO_2_ to be much more selective towards H_2_ formation than for the CO_2_RR at any applied potential. Only when a large overpotential of around −1 V is applied, can both formic acid and methanol be evolved, but low faradaic efficiency is predicted because of the more facile H_2_ formation.

## Abstract 2

Energy barriers are calculated for the electrochemical CO_2_ reduction reaction on the RuO_2_(110) surface towards methanol, methane, formic acid, methanediol, CO and the competing H_2_ formation and compared with experimental literature.

## Graphical abstract

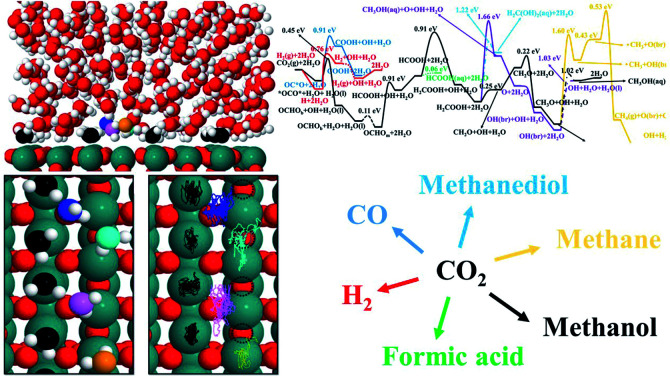

## Introduction

The electrochemical CO_2_ reduction reaction (CO_2_RR) has received a lot of attention recently, as it can be a technology to convert carbon dioxide to particular alcohols and hydrocarbons.^1–14^ The CO_2_ gas can be captured from both industrial point sources as well as from the atmosphere which would mitigate the rising levels of anthropomorphic CO_2_ emissions. Electrochemical processes are an appealing approach since the electricity can come from renewable sources (such as wind or solar energy) to make synthetic fuels for the existing infrastructure that uses fuels from fossil fuel sources. In this application, selective methanol formation would be highly desirable since it can be used directly as a liquid fuel.

The electrochemical CO_2_RR catalyzed by metallic electrodes has been studied extensively in recent years. Insight into the mechanisms or the reaction pathways for reducing CO_2_ to different products including HCOOH, CO, CH_3_OH, CH_4_, C_2_H_4_, C_2_H_5_OH *etc.*, has been obtained by both theoretical calculations^15–25^ and experimental work.^7,9^ Therein, it has been shown that previous theoretical studies using a thermochemical approach^26^ where only the adsorption free energies of intermediate species are included, are quite successful in predicting experimental overpotentials (or onset potentials).^14,16^ However, energy barriers of proton–electron transfer steps are required to elucidate the reaction mechanism and reaction pathways and capture the trends in product distribution as a function of applied potential seen in the experiments on pure metals.^21,23^

As a solvent, water usually plays an important role in various electrocatalytic reactions such as the CO_2_RR. For electrocatalytic reactions carried out in water, including the effect of solvation of reactants, intermediates, and products at the surface may be essential as the mechanism may be different.^27–30^ However, density functional theory (DFT) calculations reveal that modeling solid–liquid interfaces is complex and requires efficient functionals describing hydrogen bonding interactions.^31–34^ Despite this complexity, several groups have developed reasonable solid–liquid interface models suitable to study electrochemical processes where some electrochemical phenomena can be explained, *e.g.* catalytic trends, rates, reaction pathways and mechanisms.^23–25,35–44^

A crucial step towards a rational design of new catalysts that are selective and efficient in reducing CO_2_ to specific hydrocarbons and alcohols is to determine the detailed reaction mechanism for the process. To accomplish this, a detailed description of the electrochemical solid–liquid interface is required. Recently, it has been observed that a detailed description of the electrochemical solid–liquid interface model is successful in capturing the experimental trends of product distribution for the CO_2_RR as a function of applied potential on pure metals^23^ whereas the more approximated thermochemical model (TCM) and the implicit computational hydrogen electrode (CHE)^26^ cannot capture these trends, but do capture the overpotentials for the CO_2_RR and other electrochemical reactions quite well.^14–16,45,46^ The reason for this achievement is that the molecular level structure of water on noble transition metals is qualitatively well known and therefore realistic model systems can be used. Unfortunately, for transition metal oxide (TMO) surfaces, the molecular scale structure of water is still controversial, and therefore, realistic model systems have not been fully developed yet. However, it has been shown that TMO surfaces strongly chemisorb molecular water *via* interaction between the lone pair of the oxygen atom in the water molecule and the 5-fold coordinately unsaturated site (CUS)^28,29,47^ while on noble metal surfaces, a water bilayer is usually physisorbed on top of the metal surface.^23,33,34,37,40^ The interaction of water with a RuO_2_(110) surface has been studied experimentally by using high resolution electron energy loss spectroscopy (HREELS) and thermal desorption spectroscopy (TDS).^28^ Using HREELS, it was shown that H_2_O molecules chemisorb on Ru CUS sites through the oxygen atom of the water molecules. From HREELS and TDS results it was also found that water dissociation does not occur on the perfect RuO_2_(110) surface while a small amount of water dissociation observed in HREELS is due to some vacant Ru bridge sites.

Copper has been shown to be the only pure metal electrode catalyzing the CO_2_RR to hydrocarbons and alcohols where 15 carbon-containing products have been detected and where methane is the major product at high overpotentials (>40%).^9,48^ Other pure metal electrodes form H_2_(g), CO(g) or HCOOH(aq) as major products. For all these pure metal electrodes, methanol has been detected but in very low yields (<0.1%). Transition metal oxide surfaces, however, have been shown to catalyze the CO_2_RR to methanol in high yields (2–76%) as well as to formic acid (1–78%), depending on the applied potential and other reaction conditions, where RuO_2_-based electrodes have been studied the most.^49–53^ Methane, CO and methanediol have not been detected, except in one study where trace amounts of methane and CO were reported.^52^

Recently, RuO_2_ has been revisited experimentally for the CO_2_RR by Mezzavilla *et al.*^54^ where hydrogen gas (*via* the hydrogen evolution reaction, HER) was reported to be the main product at all applied potentials. This contradicts the previous experimental studies^49–53^ where methanol and/or formic acid were observed to be the major products. At very high overpotentials (−0.75 and −0.9 V *vs.* RHE), some trace amounts of formate and CO were observed. By alloying RuO_2_ with Ti, Cu or Sn, the faradaic efficiency of formate and CO increased to around 20% and 10%, respectively, at high overpotentials (−0.75 and −1 V *vs.* RHE). Furthermore, they show that even though RuO_2_ is not an active catalyst for the CO_2_RR to methanol, adsorbed CO is detected on the surface, when either CO or CO_2_ are introduced into the electrolyte. To the best of our knowledge, all experimental results on RuO_2_ and RuO_2_-based electrodes are summarized in Table S1 in the ESI.†

Reaction pathways have been derived from DFT calculations using the TCM and CHE models for the CO_2_RR on RuO_2_ towards formic acid, methanediol, methanol and methane and are summarized in Table 1.^55–59^ Methane and methanol are predicted to go through the same reaction pathway until the OCH_3_ intermediate is formed. The next hydrogenation results in either methanol or methane formation, but in the latter process the adsorbed oxygen atom is reduced further to water to complete the catalytic cycle. The same reaction step is predicted to be the potential limiting step (PLS) for both products, or the OCHO to HCOOH step.^55–59^ However, other elementary steps such as protonation of OCH_3_ and OH removal to form water need similar applied potentials according to thermodynamics.

**Table 1:** Reaction pathways from TCM calculations for the CO_2_RR on RuO_2_ towards formic acid, methanediol, methanol and methane formation^55–59^

Number of H^+^ + e^−^ steps								
Pathways	1	2	3	4	5	6	7	8
Formic acid	OCHO	HCOOH(aq)						
Methanediol	OCHO	HCOOH	H_2_COOH	H_2_C(OH)_2_(aq)				
Methanol	OCHO	HCOOH	H_2_COOH	CH_3_O + OH	CH_3_O + H_2_O(l)	CH_3_OH(aq)		
Methane	OCHO	HCOOH	H_2_COOH	CH_3_O + OH	CH_3_O + H_2_O(l)	CH_4_(g) + O	OH	H_2_O(l)

As described above, high yields of hydrogen,^54^ formic acid or methanol^49–53^ are detected experimentally whereas methane formation has not been reported in any studies. Clearly, the results from simple thermodynamic models (TCM and CHE) do not capture the experimental observations of these products. Therefore, a more detailed model is required, including explicit solvent effects and calculations of the proton–electron transfer energy barriers for all possible reaction steps. Within this study, the energy barriers for the HER and CO_2_RR are calculated towards various products using the (110) facet of RuO_2_ as the model system using one layer of co-adsorbed water to elucidate the reaction mechanisms to form hydrogen, methanol, methane, formic acid, methanediol and CO.

## Model systems and computational details

**Fig. 1:**
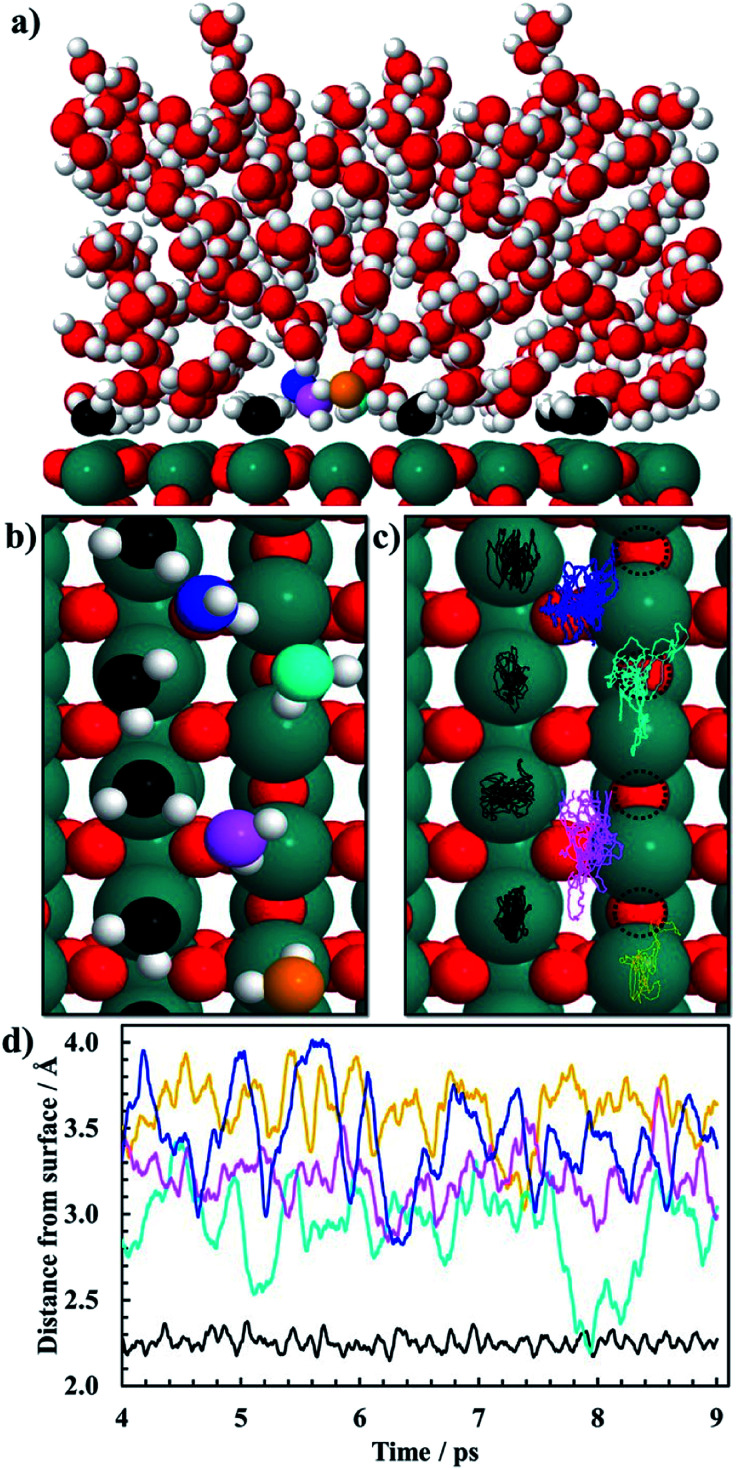
Snapshots of the last configuration of a 9 ps AIMD sampling of 84 H_2_O molecules above the RuO_2_(110) surface at 300 K showing both the (a) side view and the (b) top view. RuO_2_ is colored green for Ru and red for oxygen. H_2_O is colored red for oxygen and white for hydrogen in panel (a) except for co-adsorbed water molecules on the CUS which are colored black for oxygen and white for hydrogen. In panel (b) the same color code is used for the co-adsorbed water molecules on the CUS while water molecules in the first water bilayer are colored orange, blue, cyan and pink for oxygen atoms and white for hydrogen atoms. In panel (c) the trajectories in the *xy*-plane for the last 5 ps of the 9 ps AIMD simulations (after equilibrium was reached) corresponding to the movement of the oxygen atoms in the water molecules are shown in panel (b) where the same color code is used. Dotted-circles in panel (c) represent vacant bridge sites. In panel (d) the trajectories in the *z* direction (or the distance of the oxygen atoms of the water molecules from the Ru(110) surface) are plotted as a function of the simulation time. Same color code as in (b) and (c). Black trajectory is calculated by taking the average *z* coordinates of CUS Ru atoms at a given time and subtracted from the average *z* coordinates of the oxygen atoms in the water molecules above the CUS Ru sites for the same time. The colored trajectories are calculated by taking the average *z* coordinates of the bridge Ru atoms on the surface which are subtracted from the *z* coordinates of the oxygen atom in each of the water molecules.

*Ab initio* molecular dynamics (AIMD) simulations are carried out for the (110) facet of RuO_2_ in reduced form with five layers of water above the surface (Fig. 1a). Born–Oppenheimer AIMD simulations are carried out using the freely available program package CP2K/Quickstep.^60,61^ The density functional implementation in Quickstep is based on a hybrid Gaussian plane wave (GPW) scheme. Orbitals are described by an atom-centered Gaussian-type basis set, while an auxiliary plane wave basis is used to re-expand the electron density.^62^ Analytic Goedecker–Teter–Hutter (GTH) pseudopotentials are employed to represent the core electrons.^63,64^ The basis sets for the valence electrons consist of short-ranged (less diffuse) double-*ζ* basis functions with one set of polarization functions (DZVP).^65^ The plane wave basis for the electron density is cut off at 400 Ry while the plane wave cut off of a reference grid covered by a Gaussian is 60 Ry. All our AIMD simulations only use the Γ point of the supercell for expansion of the orbitals. The GGA–PBE functional is utilized for all AIMD calculations.^66,67^ The Grimme's D3 method is also used to include van der Waals interactions of water–water, water–Ru and water–surface oxygen.^68^ The slab consists of three atomic layers with 16 metal atoms and 32 oxygen atoms in each layer, and the exposed liquid phase is presented by 84 H_2_O molecules. The simulation box is 44 Å along the *z*-axis with a vacuum of 20 Å. The system is subject to periodic boundary conditions in all directions. Atoms in the slab along with the water molecules are allowed to reconstruct during the AIMD simulation. The simulations are performed using the Verlet algorithm with a time step of 1 fs at a temperature of 300 K within the canonical ensemble for a total simulation period of 9 ps. Generalized Langevin equation thermostats are used to enhance molecular dynamics simulations.^69,70^ The convergence of the vertical energy gap can be monitored by the time accumulative averages, as shown in Fig. S1.† Since we perform the simulation at coverage significantly close to unity, which corresponds to bulk water in contact with the RuO_2_ surface, a specific well-defined geometry is observed in the simulation during a 9 ps AIMD run of the system with 84 H_2_O molecules at room temperature. A snapshot of the equilibrated system is shown in Fig. 1a where chemisorbed water molecules decorate the CUS Ru sites (oxygen atoms of those water molecules are black).

**Fig. 2:**
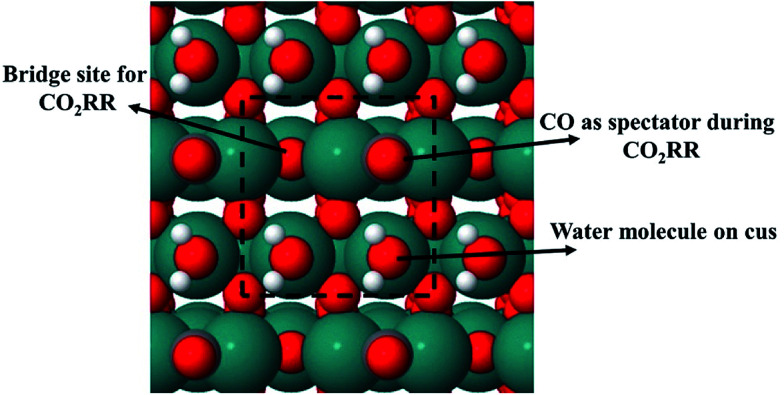
A model system of a (110) surface of RuO_2_ in its rutile crystal structure. Carbon and oxygen atoms are colored red for oxygen and grey for carbon, while hydrogen and Ru atoms are colored white for hydrogen and green for Ru. CO is a spectator species and, in all cases, located on the bridge site (oxygen vacant site) while two water molecules are located on the corresponding two CUS sites. The other oxygen vacant site (bridge site) is the only place where the CO_2_RR occurs.

To estimate the free energy of an adsorbate in the CO_2_RR and HER, static geometry optimization calculations are done using a plane-wave based pseudopotential formalism with a generalized gradient approximation (GGA) to describe the exchange–correlation effects within the BEEF–vdW functional^71^ implemented in the periodic DFT package VASP.^72,73^ All these calculations are done using a model system shown in Fig. 2 which will be further elaborated below. A plane wave basis set with a cutoff energy of 350 eV is utilized to expand the valence electron orbitals and the PAW method is used to represent the core electrons.^74^ In two separate studies, similar calculations for the CO_2_RR on RuO_2_(110) were conducted using two different cutoff values (one 350 eV (ref. 59) and the other 500 eV (ref. 56)) and they obtained similar results. Therefore, this indicates that a cutoff energy of 350 eV is accurate enough for the current study. The RuO_2_(110) slab consists of four atomic layers with four metal atoms and eight oxygen atoms in each layer. The system is subject to periodic boundary conditions in all directions. Dipole correction is applied to decouple the electrostatic interaction between the periodically repeated slabs. Atoms in the bottom two layers are fixed while the atoms in the top two layers along with adsorbed intermediates are allowed to relax during optimization. The atomic structures are determined using a 4 × 4 × 1 Monkhorst–Pack mesh for Brillouin-zone sampling until the energies are converged to within 10^−5^ eV and atomic forces drop below 0.03 eV Å^−1^. The climbing image nudged elastic band (CI-NEB)^75,76^ method is used to calculate activation energies for the HER and CO_2_RR towards different intermediates and products on the RuO_2_(110) surface.

In order to calculate the activation energy for proton–electron transfer of different intermediates for the CO_2_RR and HER on RuO_2_ with an explicit description of water, we consider only two water molecules (one monolayer, ML) on the 5-fold CUS metal site of RuO_2_ (Fig. 2). Siahrostami and Vojvodic have shown this model to be sufficient to capture the electrostatic contributions from water in the case of the oxygen evolution reaction on RuO_2_, IrO_2_ and TiO_2_.^44^ Furthermore, we justify the choice of that model system with AIMD simulations at room temperature in Fig. 1 and we apply this model when calculating activation energies towards different intermediates and products in the CO_2_RR and HER on the RuO_2_(110) electrode. When a proton–electron transfer has been carried out from one of the co-adsorbed water molecules to reduce H or CO_2_RR intermediates, an OH group is formed and adsorbed on the CUS site. This OH group should be easily protonated from the reservoir of protons in the bulk solution to regenerate co-adsorbed water molecules on the surface needed for the subsequent reduction steps.^77^ We do not calculate the energy barriers to regenerate the co-adsorbed water molecules, but we assume that the co-adsorbed water is in equilibrium with adsorbed OH and solvated proton in the bulk of electrolyte solution. We therefore only calculate the thermodynamics of the reaction:A + OH + ½H_2_(g) → A + H_2_Owhere A is an intermediate in the CO_2_RR network.

Previously, the concept of a CO spectator species in the CO_2_RR on RuO_2_(110) was introduced by Karamad *et al.*^55^ and later used by Bhowmik *et al.*^56–58^ and Tayyebi *et al.*^59^ Therefore it is assumed that the CO_2_RR takes place on a bridge site in the presence of a CO spectator which is located on every other bridge site as shown in Fig. 2. The source of adsorbed CO on the surface may therefore come from the reaction:
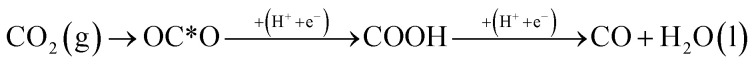


In the Results section below we indeed show that a low CO coverage will be formed on the RuO_2_(110) electrode, justifying this model system and the role of CO as a spectator species during the CO_2_RR.

## Results

AIMD simulations of equilibrated structures of 84 water molecules above the RuO_2_(110) surface are shown in Fig. 1. Snapshots of the molecular structure are shown in Fig. 1a and b, where chemisorbed water molecules decorate the CUS Ru sites (black oxygen atoms). These results are in very good agreement with the HREELS and TDS studies for adsorption of water on RuO_2_(110).^28^ To obtain some further insight into the movement of the water molecules closest to the RuO_2_(110) surface, trajectories of their oxygen atoms in the *xy*-plane and the *z*-direction are plotted in Fig. 1c and d, respectively. Fig. 1c shows that the water molecules on CUS Ru atoms stay relatively localized and remain close to their initial positions, although they exhibit some small fluctuations in the *xy*-plane. They are also found to be rather localized in the *z*-direction at around 2.25 ± 0.1 Å from the surface (black trajectory in Fig. 1d). The water molecules in the bilayer above the bridge sites of the RuO_2_(110) surface become, however, much more disordered and delocalized in the *xy*-plane as shown with the colored trajectories in Fig. 1c. They are also highly mobile in the *z*-direction and located between 2.5 and 4 Å above the surface where each molecule fluctuates by around 1 Å (colored trajectories in Fig. 1d). This shows a very weak interaction of the water molecules above the bridge Ru sites, leaving them empty to catalyze the CO_2_RR. These results support the choice of the reduced model system (Fig. 2) used to calculate the various elementary steps needed to obtain insight into the reaction mechanism.

**Fig. 3:**
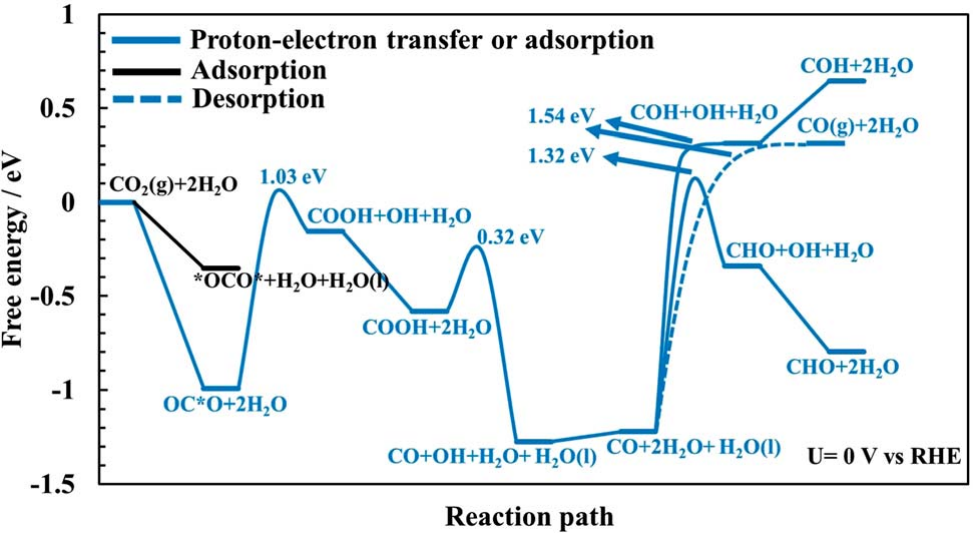
Free energy of intermediates and energy barriers for the CO_2_ reduction reaction at *U* = 0 V *vs.* RHE towards CO without any adsorbed CO as spectator species. OC*O indicates adsorbed CO_2_*via* the carbon atom while *OCO* is when CO_2_ binds to the surface through both oxygen atoms (or bidentate).

**Fig. 4:**
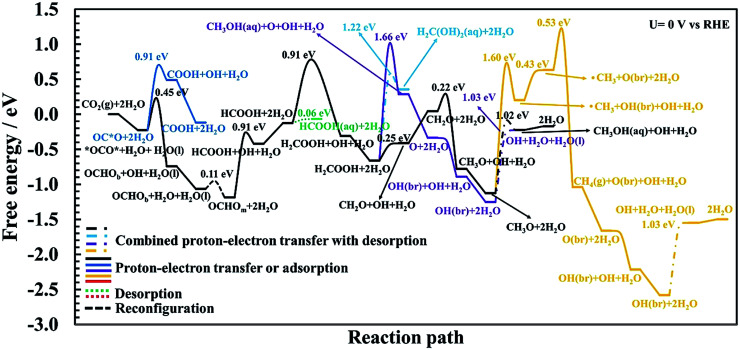
Calculated free energy and activation energy for the CO_2_RR on RuO_2_(110) at *U* = 0 V *vs.* RHE in the presence of CO as a spectator. Blue curve: pathways toward COOH. Black curve: the optimal mechanism for methanol formation. Green curve: pathway toward formic acid. Cyan curve: pathway toward methanediol. Purple curve: alternative pathway toward methanol with a lower probability compared to the optimal pathway (black curve). Orange curve: pathways toward methane. Solid lines represent proton–electron transfer barriers or adsorption processes, dash-dotted lines represent combined proton–electron transfers with desorption barriers; the dotted line is for desorption and the dashed line is for reconfiguration.

**Fig. 5:**
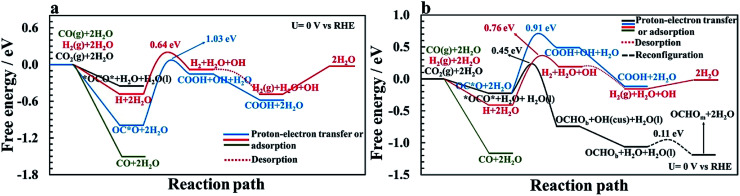
Free energy diagrams and energy barriers for hydrogen evolution reaction pathways on RuO_2_(110) at *U* = 0 V *vs.* RHE (a) without CO as a spectator and (b) with CO as a spectator (25% CO coverage). The first couple of reaction steps of the CO_2_RR (from Fig. 3 and 4) are included for comparison of these competing reactions. Adsorption free energy of CO(g) is also included for comparison.

Proton–electron transfer reaction barriers are calculated for the CO_2_RR to CO(g), HCOOH(aq), H_2_C(OH)_2_(aq), CH_3_OH(aq) and CH_4_(g) on RuO_2_(110) and compared with that of the competing H_2_(g) formation. Free energy reaction pathways along with energy barriers, *E*_a_, are presented in Fig. 3 and 4 for the CO_2_RR and in Fig. 5 for the HER at *U* = 0 V *vs.* RHE, for a range of proton–electron transfer steps, reconfiguration steps and desorption steps. The minimum energy paths (MEPs) along with configurations of the initial states, saddle points and final states for a range of possible intermediates related to the CO_2_RR and HER on RuO_2_(110) can be found in the ESI.†

In the main part of this paper it is assumed that CO_2_ is reduced on a bridge site of the surface in the presence of adsorbed CO as a spectator species which is located on every other bridge site, but water molecules are adsorbed on all CUS sites forming one ML of water coverage as explained above (Fig. 2). To start with in this paper, however, we do more detailed calculations than have been done so far in order to show that adsorbed CO is indeed a spectator species in the CO_2_RR where it neither desorbs from the surface nor reduces further to adsorbed COH or CHO species (Fig. 3). Thereafter, a low CO coverage is used as a model system to investigate the reaction pathways of the CO_2_RR towards formic acid, methanediol, methanol and methane on RuO_2_(110) in more detail (Fig. 4).

### CO formation on RuO_2_(110): why CO is a spectator species

The CO_2_RR is conducted on the RuO_2_(110) surface with 1 ML of adsorbed H_2_O molecules on the CUS Ru sites but without any adsorbed CO molecules. This represents the initial state of the surface we may expect at the time when CO_2_ is introduced in the system of the experiments. The free energies of the intermediates from CO_2_ to CO(g), CHO and COH are shown in Fig. 3 along with energy barriers of the elementary steps. We find that the way CO_2_ binds to the surface can change the reaction mechanism towards different CO_2_RR products but that is also discussed further below in relation with Fig. 4 (when we have the CO spectator species). Under these initial conditions, we obtain two different configurations for adsorption of CO_2_ on the RuO_2_(110) surface. CO_2_ can either bind where the carbon atom (OC*O) binds to a bridge site (Fig. S2a†) or where the two oxygen atoms (*OCO*) bind bidentate to a bridge site and the adjacent CUS site where one of the water molecule desorbs from the surface (Fig. S2b†). OC*O is found to be a more stable intermediate compared to *OCO* (see Fig. 3). Therefore, the next step in the CO_2_RR is to transfer a proton to the oxygen atom in OC*O to form COOH, and then further reduction of COOH leads to the formation of adsorbed CO on the bridge site. The highest energy barrier in the CO_2_RR to form adsorbed CO is 1.03 eV at 0 V *vs.* RHE (Fig. 3). Since our results predict that methanol formation needs an overpotential of around −1 V *vs.* RHE (discussed below) this energy barrier will become around 0.5 eV at that potential assuming a transfer coefficient of 0.5. Such a negative applied potential increases the chance of building up some CO coverages on the surface. This is supported in the recent experiments by Mezzavilla *et al.*^54^ where both adsorbed CO_2_ and CO were detected on the RuO_2_(110) surface at large negative potentials where it is concluded that adsorbed CO acts solely as a spectator species in the CO_2_RR.^54^ These detailed calculations, therefore, support earlier assumptions that adsorbed CO molecules may result from reduced CO_2_ molecules during the CO_2_RR experiment. There it was also shown that 25% CO coverage reproduces appropriate overpotentials for methanol formation on RuO_2_(110).^55–59^

In all of the calculation done so far for the CO_2_RR on RuO_2_(110) using the TCM model, further desorption or reduction of adsorbed CO is assumed to be slow compared to the main pathway of the CO_2_RR. Therefore, CO_2_ will be reduced all the way to *e.g.* methanol while CO remains on the surface as a spectator.^55–59^ Here we justify this assumption by calculating the energy barriers for desorption of CO to CO(g) (Fig. S6†) as well as for proton–electron transfer to CO to form either COH (Fig. S7†) or CHO (Fig. S8†) intermediates. Fig. 3 shows significant barriers for all these reaction steps (1.32–1.44 eV) predicting slow kinetics for further CO desorption/reduction which shows the validity of the assumption that has been made before in TCM simulations for the CO_2_RR on RuO_2_(110).

### Reaction pathways of the CO_2_RR on RuO_2_(110) towards formic acid, methanediol, methanol and methane

#### Adsorption of CO_2_ and proton–electron transfer barriers to either COOH or OCHO

In this section and subsequent sections, we include 25% CO coverage as a spectator species (shown in Fig. 2) and the whole reaction pathways towards different intermediates and products are shown in Fig. 4 as well as in several figures in the ESI† which will be referred to as the discussion progresses. The first step in the CO_2_RR is to adsorb CO_2_(g) on the RuO_2_(110) surface which can either bind through the carbon atom (OC*O) or with both oxygen atoms (*OCO*), referred to as a bidentate adsorption. The presence of the CO spectator on the bridge site has strong repulsion forces with the OC*O intermediate which decreases the binding energy of OC*O considerably compared with when no CO spectator is included as was shown in Fig. 3. This effect is much smaller in the case of *OCO* where the binding free energy does not change much with or without the CO spectator present. The reason for that is because *OCO* binds to one bridge site and the closest CUS site (where the H_2_O molecules desorb) and the *OCO* intermediate does not get much repulsion from the CO spectator which is located on the other bridge site. This results in the OC*O and the *OCO* intermediates having the same binding free energy in the presence of the CO spectator (Fig. 4), in stark contrast with the results obtained without any CO spectator where OC*O binds much more strongly to the surface than the *OCO* intermediate (Fig. 3). Therefore, the next step plays an important role in the selectivity towards different CO_2_RR products.

The first proton–electron transfer step can either lead to OCHO_b_ or COOH, depending on which intermediate gets protonated, *OCO* or O*CO, respectively. Here we have distinguished the bidentate configuration (OCHO_b_) where it is bonded through two oxygen atoms to a bridge site and the closest CUS site from the monodentate configuration (OCHO_m_) where it is bonded through only one of the oxygen atoms, which is discussed further below. Our calculated energy barriers in Fig. 4 show that the required barrier for protonation of *OCO* to OCHO_b_ is 0.45 eV (Fig. S9†) while for OC*O to COOH is estimated to be 0.91 eV (Fig. S4†). Therefore, OCHO_b_ is predicted to form on RuO_2_(110) instead of COOH in the presence of the CO spectator species. Here it is noted that *OCO* and OCHO_b_ are the only intermediates that bind to the surface in a bidentate form. In both cases, one of the co-adsorbed water molecules needs to be moved approximately 1.3 Å above the CUS site to provide an empty site for those species to bind to the surface. In fact, we find that the only feasible way to transfer a proton to the carbon atom in CO_2_ is that if it is adsorbed on the surface in a bidentate form (see Fig. S2b and S9†). When OCHO_b_ is formed, the next step is to rearrange OCHO_b_ to OCHO_m_ (Fig. S10†) to continue the reduction further. This is a monodentate adsorbate that binds through only one oxygen atom to the bridge site of the surface and the two water molecules are chemisorbed to the CUS sites (see final state in Fig. S10† or initial state in Fig. S11†). The energy barrier for this reconfiguration is very low (0.11 eV) and this step is slightly downhill in free energy. This rearrangement will therefore be easily overcome at room temperature.

#### Proton–electron transfer barriers from OCHO_m_ to HCOOH(aq) or H_2_COOH

The protonation of OCHO_m_ leads to adsorbed HCOOH on the surface with a barrier of 0.91 eV but a low additional barrier on top of the thermochemical barrier is observed (black curve in Fig. 4 and S11†). This barrier will be low at −1 V. The adsorbed HCOOH can easily desorb as formic acid from the surface with only a 0.06 eV barrier (green curve in Fig. 4 and S12†). Alternatively, the adsorbed HCOOH intermediate can be reduced further where the carbon atom gets protonated, resulting in adsorbed H_2_COOH with a barrier of 0.91 eV (black curve in Fig. 4 and S13†). This step has a large additional barrier since this step is already downhill at *U* = 0 V. This transition state is also the highest point on the overall energy landscape in the optimum pathway towards methanol and is predicted here to be the rate limiting step (RLS) towards methanol.

#### Proton–electron transfer barriers of H_2_COOH to CH_2_O + H_2_O(l), O + CH_3_OH(aq) or CH_2_(OH)_2_(aq)

The fourth proton–electron transfer in the CO_2_RR which is of the H_2_COOH admolecule may lead to different intermediates and products (Fig. 4). The protonation of the –OH group can take place to form an adsorbed CH_2_O intermediate and H_2_O(l) which is a part of the optimum mechanism towards methanol formation (black curve in Fig. 4), with an insignificant barrier on top of the thermodynamics (see also Fig. S14†). Another possible protonation step of H_2_COOH is to the carbon atom and to directly desorb CH_3_OH(aq) at the same time as the O–C bond is broken, which results in an adsorbed oxygen atom on the surface (Fig. S15†). This step towards methanol formation has a large energy barrier, 1.66 eV, and is not a part of the optimum pathway towards methanol which will be discussed further below. In this pathway (purple curve in Fig. 4) the adsorbed oxygen atom is protonated further towards water with no additional barriers on top of the thermodynamics (Fig. S24 and S25†). The third possible protonation step of H_2_COOH is to the oxygen atom resulting in methanediol (CH_2_(OH)_2_(aq)) formation (cyan curve, see also Fig. S16 and S17†). The energy barrier of 1.22 eV here at *U* = 0 V will be surmountable at −1 V applied potential, but the selectivity will be much more favored towards the CH_2_O intermediate which can be reduced further to methanol. This is in agreement with several of the reported experiments where high efficiency towards methanol is reported whereas methanediol has not been detected in any of those.

#### Proton–electron transfer barriers of CH_2_O to either methanol or methane

The protonation of CH_2_O leads to adsorbed CH_3_O on the surface (black curve in Fig. 4). The calculated activation energy for this reaction is estimated to be 0.22 eV (see also Fig. S18†). When the CH_3_O intermediate is formed on the surface, it may be protonated to form methanol which desorbs directly from the surface with an energy barrier of 1.02 eV, but has a small additional barrier above the thermochemical barrier which will be low at −1 V applied potential (see also Fig. S19†). This is therefore the PLS for methanol formation.

In order to investigate methane formation, several possible pathways and mechanisms are considered. The mechanism with the lowest overall barrier is considered first but alternative mechanisms are explored below. The protonation of adsorbed CH_3_O may also result in adsorbed CH_3_OH and subsequent cleavage of the O–C bond in HO–CH_3_, resulting in a planar ˙CH_3_ radical desorbed slightly from the surface and leaving an OH species adsorbed at the bridge site on the surface, OH(br). This mechanism has a significantly higher barrier than when directly desorbing methanol as discussed above, with an energy barrier of 1.60 eV, and a considerably large additional barrier above the thermodynamics (yellow curve in Fig. 4 and S21†). The subsequent reduction of this ˙CH_3_ radical to desorbed CH_4_(g) has also a significantly large overall barrier as described further here. After the planar ˙CH_3_ radical is formed, the hydrogen atom in the adsorbed OH(br) intermediate is transferred back to adsorbed OH on the CUS site, forming co-adsorbed water on the surface (yellow curve in Fig. 4 and S22†). The additional barrier is insignificant, but the thermochemical barrier is uphill by 0.43 eV. This co-adsorbed water then transfers a proton–electron to the ˙CH_3_ radical to form methane. The additional barrier is moderately high or 0.53 eV (Fig. S23†) but the overall barrier for this mechanism is large, or around 2.3 eV (yellow curve in Fig. 4). The difference in the overall barrier height between methanol formation (1.02 eV) and methane formation *via* this mechanism (2.3 eV) results in a large difference in selectivity towards these products. This difference in energy barriers between these two products corresponds to around a 10^22^ order of magnitude difference in rates at *U* = 0 V and under standard conditions.^23^

Since the mechanism of methane formation is a crucial step in the overall work and the mechanism presented above resulted in an overall high energy barrier it requires further investigation. The proton transfer can also be to the carbon atom of the O–CH_3_ admolecule to form a desorbed CH_4_(g) molecule directly (not shown in Fig. 4, but shown Fig. S20†). The calculation of the minimum energy pathway for this mechanism results in the O–C bond in the adsorbed O–CH_3_ being broken first, resulting in this same planar ˙CH_3_ radical above the surface which is then protonated to form methane. The calculated activation energy for this combined mechanism is 2.77 eV, with a large additional barrier of more than 2 eV. Comparing the overall barrier of 2.77 eV for this mechanism with the one presented above (2.3 eV) shows that a lower barrier can be achieved by forming first methanol and then breaking the O–C bond before the ˙CH_3_ radical is reduced. However, in both cases, an insignificant rate of methane formation is predicted at room temperature compared to the rate of methanol formation.

Because in both mechanisms presented above for methane formation a ˙CH_3_ radical is first formed before it can be reduced to a CH_4_(g) molecule, yet another alternative mechanism is considered. Here we consider whether a water molecule in the first bilayer can assist in this reaction. The proton is shuttled from the co-adsorbed water to this additional water molecule *via* a Grotthuss mechanism and transferred to the methyl group of the O–CH_3_ admolecule to form methane directly. A mechanism like this would bypass forming the ˙CH_3_ radical first and could lower the overall barrier because this concerted mechanism should weaken the O–CH_3_ bond. During simulation of this reaction path, however, a proton–electron transfer from the co-adsorbed water to the oxygen atom of the CH_3_O admolecule results in an initial formation of a methanol molecule that desorbs from the surface instead of favoring the initially set reaction path. Therefore, it rather favors the mechanism presented initially having a barrier around 2.3 eV and the one included in Fig. 4. This shows that the proton–electron transfer is carried out with the co-adsorbed water rather than from a water molecule in the first bilayer.

The high barriers for methane formation presented above provide insight and reasoning as to why methane is not detected experimentally despite the fact that the simple TCM calculations predict the same overpotentials and PLS for both methane and methanol formation as introduced earlier in this work. It should be noted that we predict here the OCH_3_ to CH_3_OH(aq) step to be the PLS whereas the HCOOH to H_2_COOH is the RLS for methanol formation as explained above.

#### Hydrogen evolution

As described above, H_2_ evolution has recently been concluded to be the dominant reaction on RuO_2_ electrodes,^54^ contradicting all previous reports where methanol and/or formic acid were identified as the major products.^49–52^ Therefore, the mechanism of the HER is calculated on the same model systems as presented above for the CO_2_RR in order to determine the selectivity between the HER and CO_2_RR. However, only using two co-adsorbed water molecules on the CUS results in a very high energy barrier (around 2 eV) for transferring the proton from the co-adsorbed water molecule to the vacant bridge site (Volmer reaction). This can be attributed to a relative long distance for the proton transfer in this case compared to when CO_2_RR intermediates are adsorbed and reduced on the vacant bridge site presented above. Therefore, we include an additional water molecule from the first bilayer to assist in this reaction, similar as was done in one of the methane formation mechanisms presented above. The proton is then shuttled from the co-adsorbed water to this additional water molecule *via* a Grotthuss mechanism and transferred to the bridge vacant site and the calculated energy barrier for this reaction is 0.46 eV at *U* = 0 V *vs.* RHE (Fig. S26†). Adding protons to the active sites on the RuO_2_(110) surface is therefore concluded to be facile at reducing potentials. For the H_2_ formation step presented below we find that when the bridge site is already occupied by a hydrogen adatom the presence of this additional water molecule has a minor effect on the energy barrier, changing it by only +0.04 and −0.15 eV, without and with a CO spectator, respectively, as compared to when only using the two co-adsorbed water molecules. Therefore, in order to maintain constancy, proton–electron transfer barriers for the H_2_ formation step are also carried out using the three water molecule model system.

Fig. 5a shows the comparison of the competing HER with the CO_2_RR on a clean RuO_2_(110) surface at *U* = 0 V *vs.* RHE. The adsorption free energy of OC*O is around −1 eV and binds stronger to the surface than a H adatom of around −0.5 eV where both intermediates adsorb to a bridge vacant site. Since in a CO_2_-saturated solution the proton concentration is much higher than the CO_2_ concentration it is reasonable to assume that these sites will have a higher coverage of H than adsorbed CO_2_. However, when CO_2_ comes into the double layer it will adsorb stronger than protons. Since the applied potential affects the proton adsorption much more strongly than the CO_2_ adsorption, these results indicate that the adsorption free energies of H and CO_2_ become equal at −0.5 V *vs.* RHE. After adsorption of hydrogen on the surface, the next step is to protonate hydrogen using co-adsorbed water and produce adsorbed H_2_ on the surface. The calculated activation energy for this step is estimated to be 0.64 eV at *U* = 0 V. In the next step, H_2_ leaves the surface without any energy barrier. This is compared with the CO_2_RR pathway towards forming adsorbed CO on the surface, where the highest barrier is 1.03 eV. These results indicate that the HER is much more facile than the CO_2_RR on the clean RuO_2_(110) surface at reducing potentials, but when very negative potential is applied, some CO_2_ will be reduced to adsorbed CO, in agreement with recent experimental results.^54^ Another way to introduce adsorbed CO as a spectator species on the surface is to include some CO gas into the electrolyte before CO_2_RR experiments. Fig. 5a shows that CO adsorbs much stronger to the surface than either protons or CO_2_.

The same competing processes of the HER and CO_2_RR are compared in Fig. 5b when a CO spectator is adsorbed on the surface. The presence of CO on the surface destabilizes the binding free energy of hydrogen slightly, or to a value of around −0.4 eV. While the adsorption free energy of *OCO* is only slightly affected by the presence of CO, the adsorption of OC*O is largely affected, and binds weaker than H. This results in even higher possibilities of the catalytic sites being occupied with H rather than CO_2_, especially when reducing potentials are applied. The energy barrier for H_2_ formation increases to around 0.76 eV at 0 V *vs.* RHE with the CO spectator (Fig. S28†), compared to a barrier of around 0.64 eV without CO (Fig. S27†). The H_2_ formation steps are concluded to be the RLS for the HER as the energy barriers are higher than for the Volmer step presented above. Fig. 5b shows that the competing energy barrier for the formate pathway in the CO_2_RR is only 0.45 eV at 0 V *vs.* RHE. As shown in Fig. 4, the PLS for formic acid formation is the reduction of the formate intermediate which requires −0.9 V. At these reducing potentials, most of the bridge sites will be covered with protons, resulting in much higher rates of the HER than the CO_2_RR, in good agreement with recent experimental findings.^54^

## Discussion

In this work, the proton–electron transfer barriers can be classified into four groups:

1. Proton–electron transfer to oxygen (*e.g.* O–CH–O to O–CH–OH)

2. Proton–electron transfer to the hydroxyl group (*e.g.* O–CH_2_–OH to O–CH_2_ + H_2_O(l))

3. Proton–electron transfer to carbon (*e.g.* O–C–O to O–CH–O)

4. Proton–electron transfer to hydrogen

Overall, the proton–electron transfer steps presented in this paper show that attacking the –O or –OH groups of CO_2_RR intermediates results in low additional barriers on the order of 0.0 to 0.3 eV while attacking the carbon atom results in higher additional barriers. For highly exergonic reactions they are around 0.2 to 0.5 eV. For the reaction having around thermoneutral or endergonic reaction free energies, the additional energy barrier is around 0.5 to 0.9 eV, except in the case of methane formation, which is over 2 eV. This is not surprising since oxygen is more electronegative than carbon which should favor a proton transfer. Finally, in the case of adsorbed H, our results show low additional barriers, or 0.34 and 0.15 eV, without and with a CO spectator, respectively. The energy barriers presented in this work are summarized in Table 2 where they are classified based on which atom of the CO_2_RR or H intermediates protonation takes place.

**Table 2:** Calculated reaction energies (Δ*E*), energy barriers (*E*_a_) and additional energy barriers on top of thermodynamics for several possible intermediates and products for the CO_2_RR and HER on RuO_2_(110). Here, S stands for the CO spectator. All energies are in eV

Reactions	Classification	Δ*E*	*E*_a_	Additional barrier
CO_2_ + H_2_O → COOH + OH	Transfer to O	0.78	0.88	0.10
CO_2_ + H_2_O → OCHO_b_ + OH (S)	Transfer to C	−0.87	0.45	0.45
OCHO_b_ → OCHOm (S)	Reconfiguration	−0.11	0.11	0.11
OCHO + H_2_O → HCOOH + OH (S)	Transfer to O	0.72	0.86	0.14
HCOOH → HCOOH(aq) (S)	Desorption	0.20	0.20	0.00
HCOOH + H_2_O → H_2_COOH + OH (S)	Transfer to C	−0.33	0.91	0.91
H_2_COOH + H_2_O → H_2_C(OH)_2_ + OH (S)	Transfer to O	0.52	0.72	0.20
H_2_C(OH)_2_ → H_2_C(OH)_2_(aq) (S)	Desorption	0.60	0.60	0.00
H_2_COOH + H_2_O → CH_2_O + H_2_O(l) + OH (S)	Transfer to OH	0.78	0.78	0.00
H_2_COOH + H_2_O → CH_3_OH(aq) + O + OH (S)	Transfer to C	1.13	1.66	0.53
CH_2_O + H_2_O → CH_3_O + OH (S)	Transfer to C	−0.91	0.22	0.22
CH_3_O + H_2_O → CH_3_OH(aq) + OH (S)	Transfer to O	0.94	1.06	0.12
CH_3_O + H_2_O → ˙CH_3_ + OH(br) + OH(CUS) (S)	Transfer to O	1.50	1.97	0.47
OH(br) + OH(CUS) → O(br) + H_2_O(CUS) (S)	Reconfiguration	0.74	0.74	0.00
˙CH_3_ + O(br) + H_2_O → CH_4_(g) + O(br) + OH(CUS) (S)	Transfer to C	−1.42	0.53	0.53
CH_3_O + H_2_O → CH_4_(g) + O + OH (S)	Transfer to C	0.64	2.77	2.13
O + H_2_O → OH + OH (S)	Transfer to O	−0.62	0.00	0.00
COOH + H_2_O → CO + H_2_O(l) + OH	Transfer to OH	−0.33	0.32	0.32
CO + H_2_O → COH + OH	Transfer to O	1.57	1.57	0.00
CO + H_2_O → CHO + OH	Transfer to C	0.97	1.41	0.44
CO → CO(g)	Desorption	1.98	1.98	0.00
H + H_2_O → H_2_(g) + OH	Transfer to H	0.30	0.64	0.34
H + H_2_O → H_2_(g) + OH (S)	Transfer to H	0.61	0.76	0.15

In the case of pure metal electrodes, the reaction pathways are very different depending on whether a detailed solvation and kinetic model is used or a thermochemical one.^23^ Despite a much more detailed modeling of the reaction paths presented in this work for RuO_2_(110) than in previous studies using the TCM and CHE models,^55–59^ both methods predict the same adsorbed intermediates between proton–electron transfer steps, except in one case. Table 3 summarizes the theoretical reaction pathways based on our current work for the CO_2_RR towards methanol and methane and should be compared with the results in Table 1 which summarizes the pathways predicted by the TCM–CHE models. After the fourth proton–electron transfer step we predict with the detailed modeling that the H_2_COOH admolecule is reduced to the CH_2_O intermediate on the surface and an H_2_O(l) molecule that desorbs from the surface (Table 3), whereas the simpler TCM model predicts the CH_3_O + OH intermediates on the surface (Table 1). This is however not because of the negligible kinetic barrier we find for this step, but because of the presence of co-adsorbed water on the surface that imposes the formation of adsorbed CH_2_O and H_2_O(l) instead of forming the CH_3_O and OH intermediates. The CH_3_O and OH intermediates will be formed when CH_2_O is protonated further by co-adsorbed water (Fig. 4 and Table 3).

**Table 3:** Reaction pathways towards methanol and methane formation based on our current study

Pathways	1	2	3	4	5	6	7	8
Methanol	OCHO	HCOOH	H_2_COOH	CH_2_O + H_2_O(l)	CH_3_O	CH_3_OH(aq)		
Methane	OCHO	HCOOH	H_2_COOH	CH_2_O + H_2_O(l)	CH_3_O	CH_4_(g) + O	OH	H_2_O(l)

Finally, we address the question of why Cu is more selective towards methane than methanol while the opposite trend is observed and predicted here on RuO_2_; *i.e.* more selective towards methanol than methane. There is a fundamental difference between these two types of catalysts which alters the binding configurations of the CO_2_RR intermediates, which changes the selectivity. While all intermediates bind through the carbon atom on the Cu(111) surface,^23^ they all bind through their oxygen atom(s) on the RuO_2_(110) surface. As an example, on Cu(111) the first two intermediates are predicted to be COOH and CO where both bind to the surface through the carbon atom, while OCHO and HCOOH are predicted to be the first two intermediates on RuO_2_(110), both binding through their oxygen atom(s). This is presumably because of the oxygen vacant bridge sites on RuO_2_(110), which tend to bind intermediates through their oxygen atoms rather than through their carbon atom. Furthermore, we observe that when an intermediate binds to the surface through an oxygen atom, a very high activation energy is needed to break the O–C bond; as shown in the case of O–CH_3_ reduction to O + CH_4_ in Table 2. Therefore, O–CH_3_ is much rather reduced to methanol than methane on RuO_2_. In contrast, when intermediates bind through the carbon atom to the surface, breaking the C–O bond requires a very low activation energy, as shown in the case of COOH reduction to CO + H_2_O in Table 2 for RuO_2_ and has been shown previously for Cu(111).^23^ This is why Cu is more selective towards methane than methanol where all intermediates bind through the carbon atom.

## Conclusions

In this work, we calculate proton–electron transfer energy barriers for the CO_2_RR and HER on RuO_2_(110) using 1 ML of co-adsorbed water molecules on the CUS and low CO coverages on the surface to gain further insight into the reaction mechanisms towards hydrogen, methanol, methane, methanediol, formic acid and CO evolution. We obtained two substantial additional energy barriers on top of the thermodynamics. One is related to a proton–electron transfer to the O–CH–OH species to form O–CH_2_–OH with an additional energy barrier of 0.91 eV which is the RLS towards methanol and methanediol. The highest energy barrier we find towards methanol is, however, 1.02 eV, for the reduction of the OCH_3_ intermediate, but since the barrier mainly comes from the thermodynamics of that reaction step it is easily surmounted at room temperature at the required overpotential for methanol formation which is calculated to be around −1 V *vs.* RHE. The other substantial additional energy barrier we find in this work is related to the protonation of the O–CH_3_ intermediate to form methane with an overall barrier of 2.3 eV or more. A few mechanisms are considered where they all resulted in having more than one elementary step. In all cases, high barriers for breaking the O–C bond are found to be limiting. These results show clearly from the energy barriers why methanol can be formed on RuO_2_ electrodes at high overpotentials while methane formation seems impossible. The calculations predict, however, that hydrogen is the main product at all potentials which is consistent with the most recent experimental results.

Generally, we find that the additional energy barriers on top of the thermodynamics for proton–electron transfer to –O and –OH groups in CO_2_RR intermediates are low. This indicates that these elementary steps are easily surmountable at the overpotential required for the reaction and they are not kinetically limited. In contrast, proton–electron transfer steps to the carbon atom in CO_2_RR intermediates and for O–C scission steps are predicted to be kinetically limited in some cases even if the thermodynamics are favorable. Furthermore, we find that the required barriers to desorb CO from the RuO_2_(110) surface or to further reduce it to COH or CHO are relatively high and this supports the hypothesis regarding CO as a spectator species on RuO_2_ electrodes.

## Conflicts of interest

There are no conflicts to declare.

## Supplementary Material

SC-011-D0SC01882A-s001
