# Supplementary material for: Why do RuO2 electrodes catalyze electrochemical CO2 reduction to methanol rather than methane or perhaps neither of those?
Source: Chem Sci. 2020 Jul 30;11(35):9542–53. doi: 10.1039/d0sc01882a (PMC8161686; doi:10.1039/d0sc01882a)
Supplement: SC-011-D0SC01882A-s001 [file SC-011-D0SC01882A-s001.pdf]

Electronic Supplementary Material (ESI) for Chemical Science.
This journal is © The Royal Society of Chemistry 2020

Supporting Information

Why RuO_2 electrodes catalyze electrochemical CO_2 reduction to methanol rather than methane; or perhaps neither of those?

Ebrahim Tayyebi[1], Javed Hussain[1], Egill Skúlason[1,2,*]

[1]Science Institute, University of Iceland, VR-III, 107 Reykjavík, Iceland

[2]Faculty of Industrial Engineering, Mechanical Engineering and Computer Science, University of Iceland, VR-III, 107 Reykjavík, Iceland

*Corresponding Author: egillsk@hi.is

Table S1. Recent experimental results for electrochemical CO_2 reduction reaction on RuO_2 and RuO_2-based catalysts.

Electrode	CO_2 saturated electrolyte	pH	Potential (V)	Time (min)	Faradic efficiency (%)				
					CH_3OH	HCOOH	H_2	CO	CH_4
RuO_2 (35)/TiO_2 (65) [1]	0.05 M Hg_2SO_4	1.2	-0.15 vs RHE	>2400	24	2	---	---	---
Ag-doped RuO_2 (35) / TiO_2 (65) [1]	0.05 M Hg_2SO_4	1.2	-0.15 vs RHE	---	2	78	---	---	---
Rh_2O_3 (20)/ TiO_2 (80) [1]	0.05 M Hg_2SO_4	1.2	-0.15 vs RHE	---	5	---	---	---	---
RuO_2 (25) / MoO_2 (30) / TiO_2 (45) [1]	0.05 M Hg_2SO_4	1.2	-0.15 vs RHE	---	12	<1	---	---	---
RuO_2 (20) / Co_3O_4 (10) / SnO_2 (8) / TiO_2 (62) [1]	0.05 M Hg_2SO_4	1.2	-0.15 vs RHE	---	7	18	---	---	---
RuO_2/TiO_2 [1]	0.2 M Na_2SO_4	4.0	Water reduction potential	>2400	76	---	---	---	---

RuO_2/TiO_2 [1]	0.2 M phosphate buffer	5.2	Water reduction potential	>2400	35	---	---	---	---
RuO_2/TiO_2 [1]	0.5 M $KHCO_3$	7.6	Water reduction potential	>2400	5	---	---	---	---
RuO_2/TiO_2 [1]	0.2 M Na_2SO_4	4.0	Water reduction potential	>2400	53	---	---	---	---
Cu-doped RuO_2 (75) / TiO_2 (25) [2]	0.5 M $KHCO_3$	7.2 to 7.3	-0.15 vs RHE	---	29.8	4.2	---	---	---
RuO_2 [3]	0.5 M $NaHCO_3$	8.3	-0.15 vs RHE	480	17.2	---	---	---	---
Cu-doped RuO_2 [3]	0.5 M $NaHCO_3$	8.3	-0.15 vs RHE	480	41.3	---	---	---	---
Cd-doped RuO_2 [3]	0.5 M $NaHCO_3$	8.3	-0.15 vs RHE	480	38.2	---	---	---	---
RuO_2 – coated boron doped diamond [4]	0.05 M Hg_2SO_4	5.9	0.21 vs RHE	206	8.12	32.66	39.88	0.005	0.11
RuO_2 – coated boron doped diamond [4]	0.05 M Hg_2SO_4	7.3	0.09 vs RHE	356	4.77	37.45	44.85	0.003	0.12
RuO_2/TiO_2 nano particles composite [5]	0.5 M $NaHCO_3$	8.5	-0.15 vs RHE	120	40.2	---	---	---	---
RuO_2/TiO_2 nano tubes composite [5]	0.5 M $NaHCO_3$	8.5	-0.15 vs RHE	120	60.5	---	---	---	---
RuO_2 [6]	0.1 M $KHCO_3$	6.8	-0.50 vs RHE	>900	0.00	0.00	>95	0.00	0.00

RuO_2 [6]	0.1 M $KHCO_3$	6.8	-0.90 vs RHE	>900	0.00	<0.2	>90	0.1<	0.00
RuO_2 (50)/TiO_2 (50) [6]	0.1 M $KHCO_3$	6.8	-0.75 vs RHE	>900	0.00	0.5<	>95	0.5<	0.00
RuO_2 (25)/TiO_2 (75) [6]	0.1 M $KHCO_3$	6.8	-0.75 vs RHE	>900	0.00	0.00	>90	1.00<	0.00
Cu-doped RuO_2 (25)/TiO_2 (75) [6]	0.1 M $KHCO_3$	6.8	-0.75 vs RHE	>900	0.00	5.00<	83<	12.5<	0.00
RuO_2 (25)/SnO_2 (75) [6]	0.1 M $KHCO_3$	6.8	-1.00 vs RHE	>900	0.00	3.00<	95<	2.00<	0.00
RuO_2 (10)/SnO_2 (90) [6]	0.1 M $KHCO_3$	6.8	-1.00 vs RHE	>900	0.00	3.00<	94<	6.00<	0.00

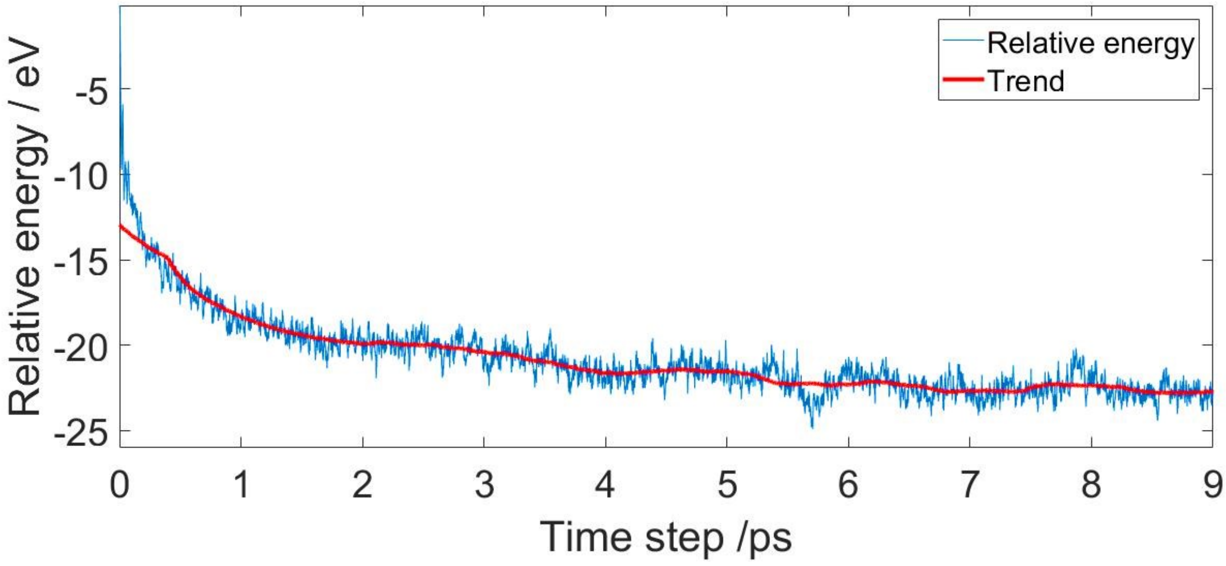

Figure S1:Relative energy as a function of elapsed time from *ab initio* molecular dynamic simulation at 300 K of 84 H_2O molecules on top of a RuO_2(110) surface.

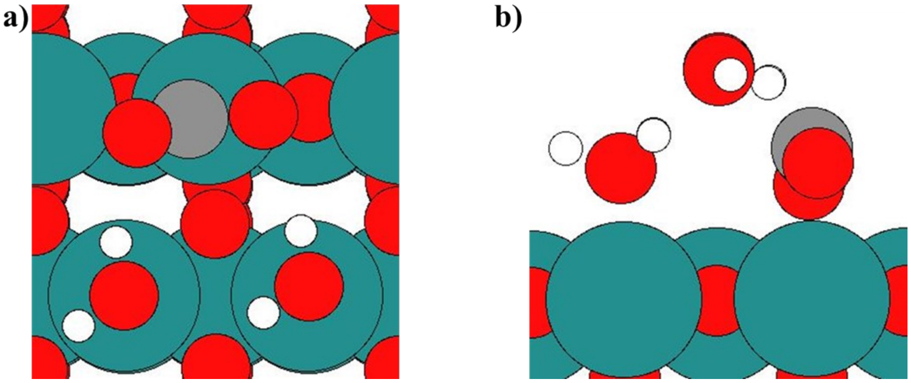

Figure S2. a) top view of O*CO where the carbon atom binds to a bridge site and b) side view of *OCO* where two oxygen atoms bind to a bridge site and the adjacent CUS.

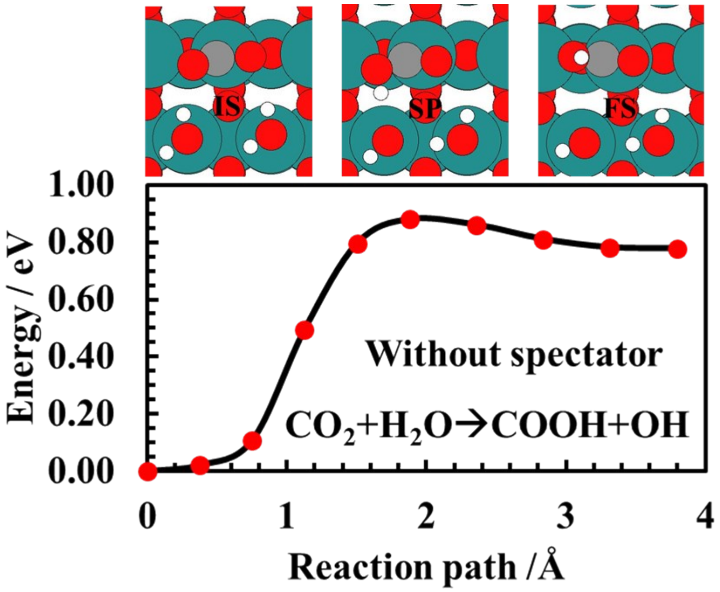

Figure S3. DFT calculated barrier for CO_2 protonation to COOH on RuO_2(110) surface. Similar value for activation energy of CO_2 to COOH has been obtained on RuO_2(110) when there is one CO as spectator on the surface (figure S2). (IS) initial state, (SP) saddle point and (FS) final state are shown on the top of the figure.

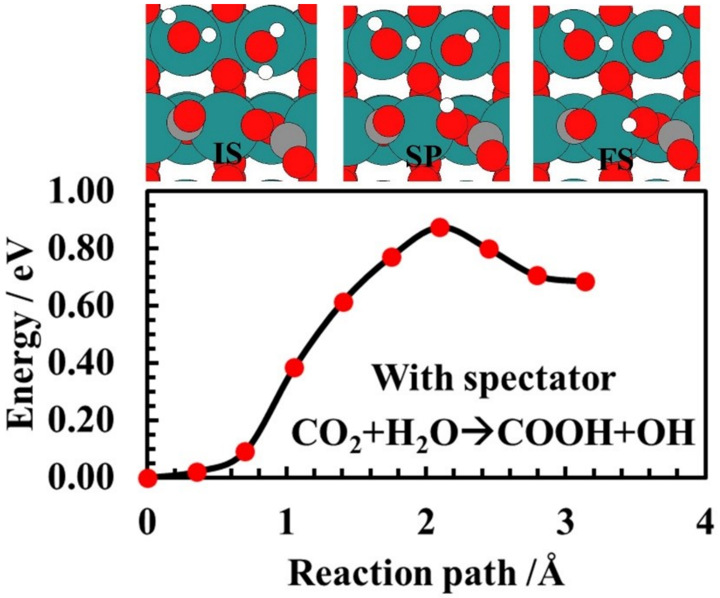

Figure S4. DFT calculated barrier for CO_2 protonation to COOH on $RuO_2(110)$ surface where CO spectator is included. (IS) initial state, (SP) saddle point and (FS) final state are shown on the top of the figure.

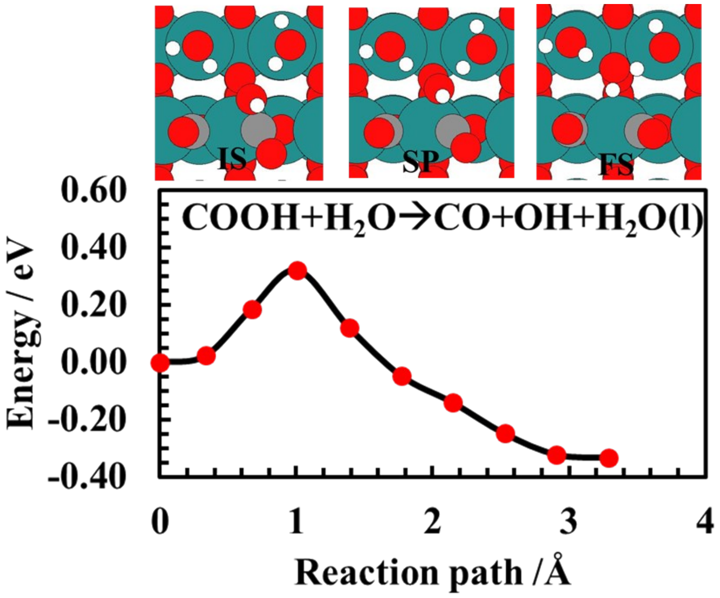

Figure S5. DFT calculated barrier for COOH protonation to CO and H_2O on $RuO_2(110)$ surface. Similar value for activation energy is expected to be obtained on $RuO_2(110)$ surface even without the presence of spectator. (IS) initial state, (SP) saddle point and (FS) final state are shown on the top of the figure.

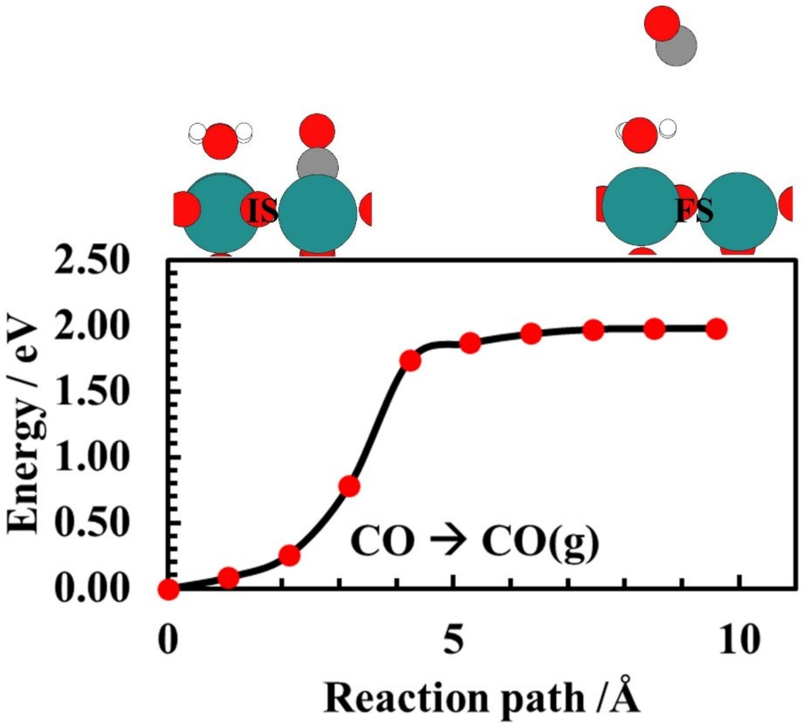

Figure S6. DFT calculated barrier for CO desorption on RuO_2(110) surface. (IS) initial state and (FS) final state are shown on the top of the figure.

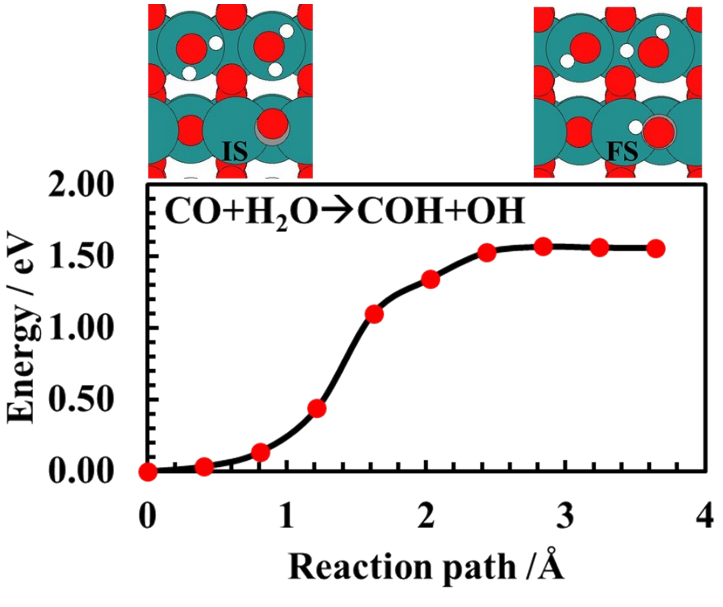

Figure S7. DFT calculated barrier for CO protonation to COH on RuO_2(110) surface. (IS) initial state and (FS) final state are shown on the top of the figure. The proton is transferred to CO from the adjacent H_2O molecule using a concerted Grotthuss mechanism of proton transfer from the next H_2O molecule on the CUS.

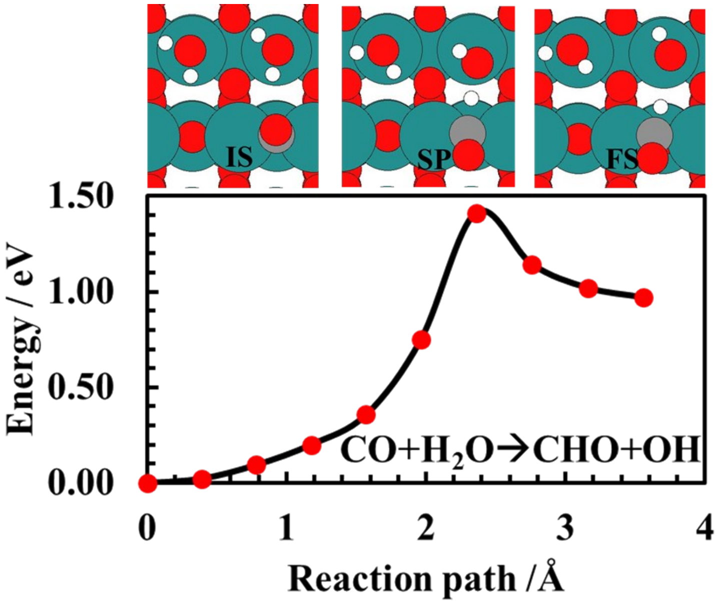

Figure S8. DFT calculated barrier for CO protonation to CHO on RuO_2(110) surface. (IS) initial state, (SP) saddle point and (FS) final state are shown on the top of the figure.

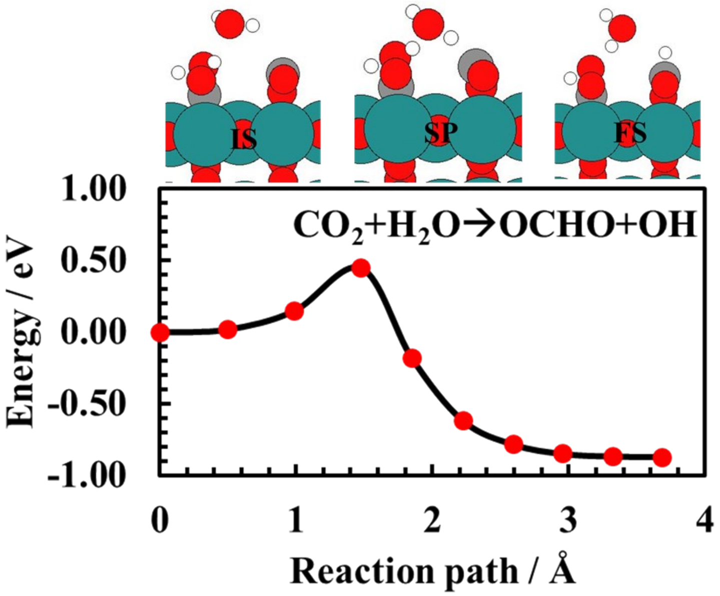

Figure S9. DFT calculated barrier for CO_2 protonation to $OCHO_b$ in a bidentate adsorption configuration on RuO_2(110) surface. (IS) initial state, (SP) saddle point and (FS) final state are shown on the top of the figure.

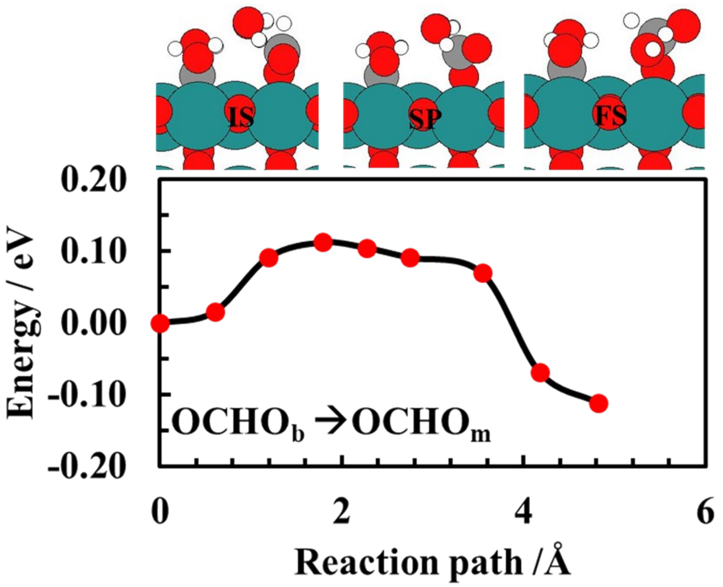

Figure S10. DFT calculated barrier for OCHO reconfiguration from bidentate configuration $OCHO_b$ to monodentate configuration $OCHO_m$ on RuO_2(110) surface. (IS) initial state, (SP) saddle point and (FS) final state are shown on the top of the figure.

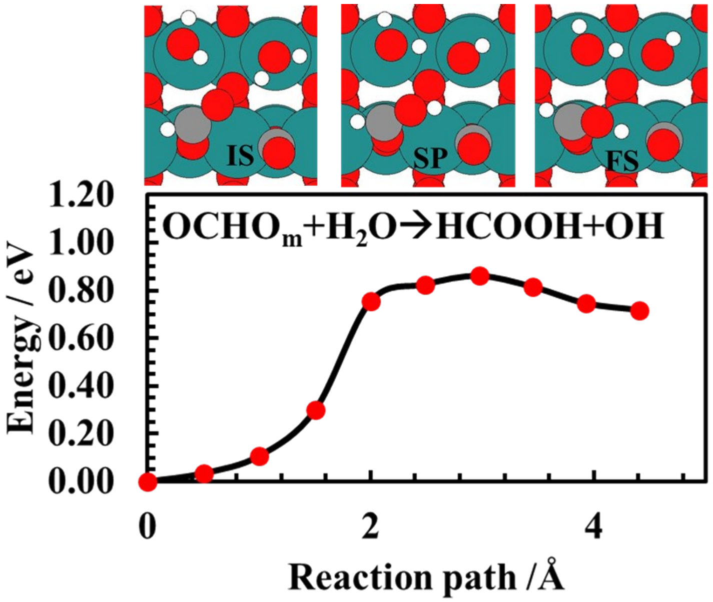

Figure S11. DFT calculated barrier for $OCHO_m$ protonation to HCOOH on RuO_2(110) surface. (IS) initial state, (SP) saddle point and (FS) final state are shown on the top of the figure.

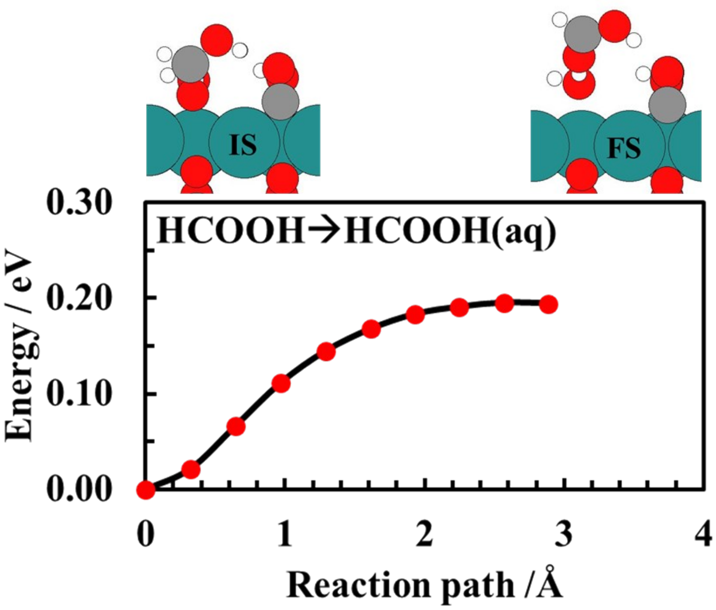

Figure S12. DFT calculated barrier for formic acid desorption on RuO_2(110) surface. (IS) initial state and (FS) final state are shown on the top of the figure.

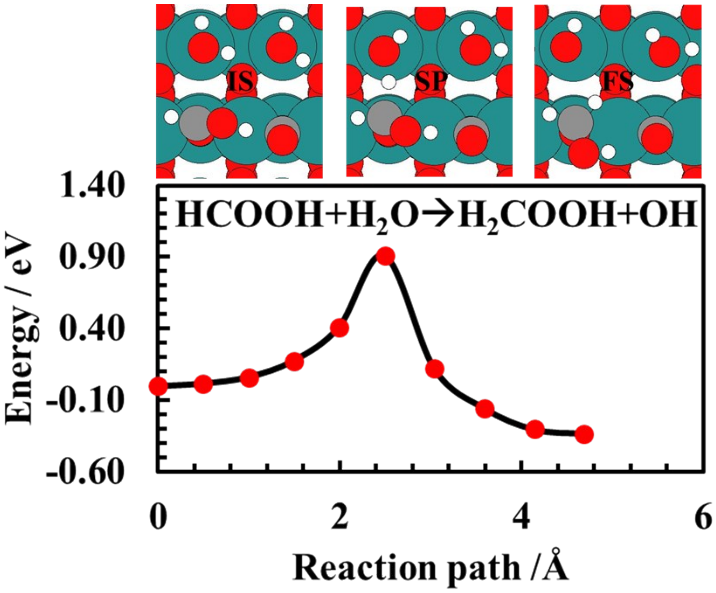

Figure S13. DFT calculated barrier for HCOOH protonation to H_2COOH on RuO_2(110) surface. (IS) initial state, (SP) saddle point and (FS) final state are shown on the top of the figure.

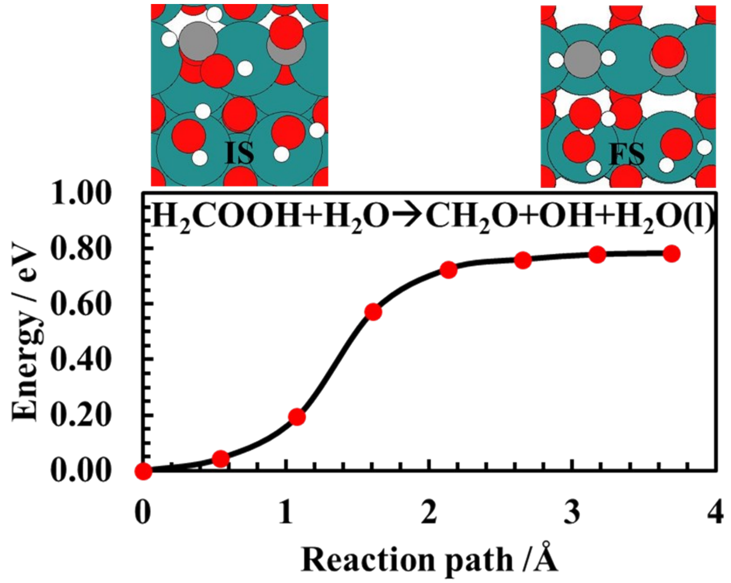

Figure S14. DFT calculated barrier for H_2COOH protonation to CH_2O and H_2O on $RuO_2(110)$ surface. (IS) initial state and (FS) final state are shown on the top of the figure.

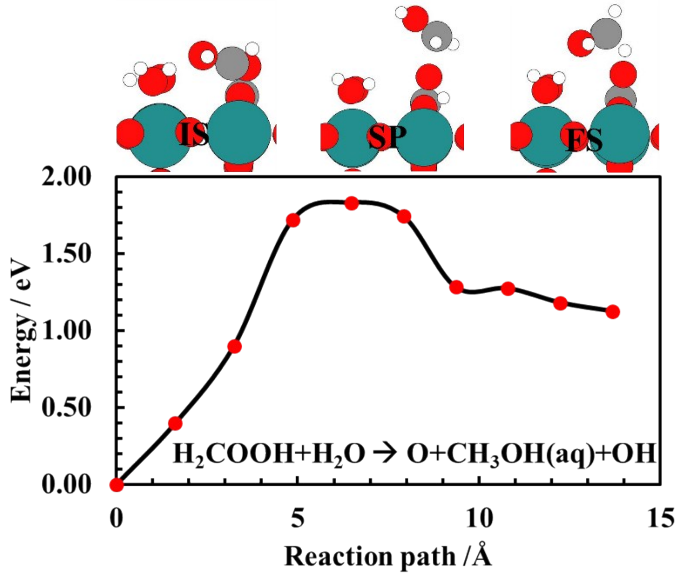

Figure S15. DFT calculated barrier for H_2COOH protonation to $H_2C(OH)_2$ on $RuO_2(110)$ surface. (IS) initial state, (SP) saddle point and (FS) final state are shown on the top of the figure.

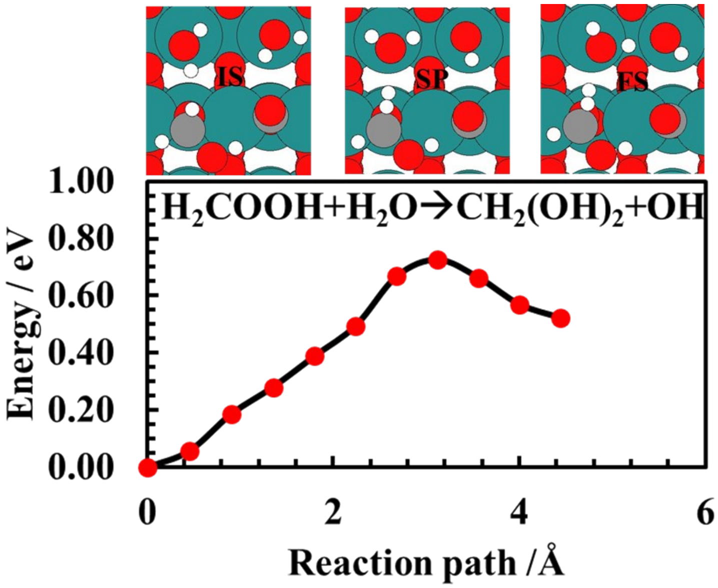

Figure S16. DFT calculated barrier for H_2COOH protonation to $H_2C(OH)_2$ on $RuO_2(110)$ surface. (IS) initial state, (SP) saddle point and (FS) final state are shown on the top of the figure.

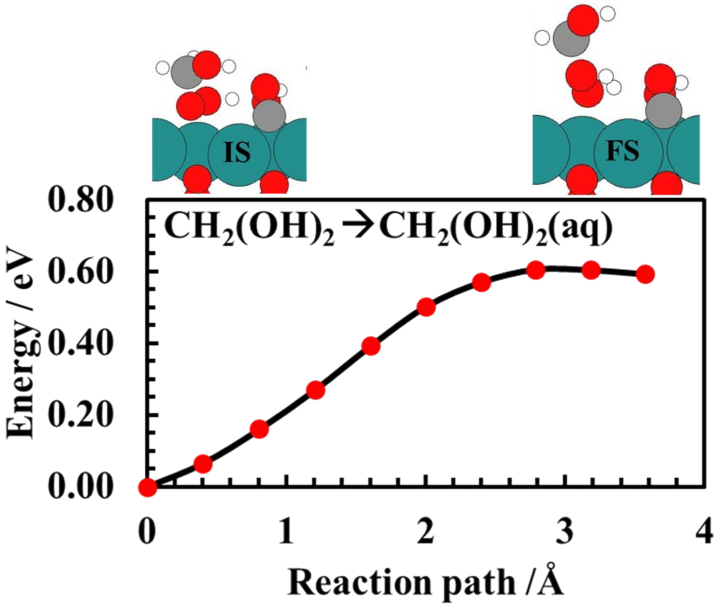

Figure S17. DFT calculated barrier for methanediol desorption on $RuO_2(110)$ surface. (IS) initial state and (FS) final state are shown on the top of the figure.

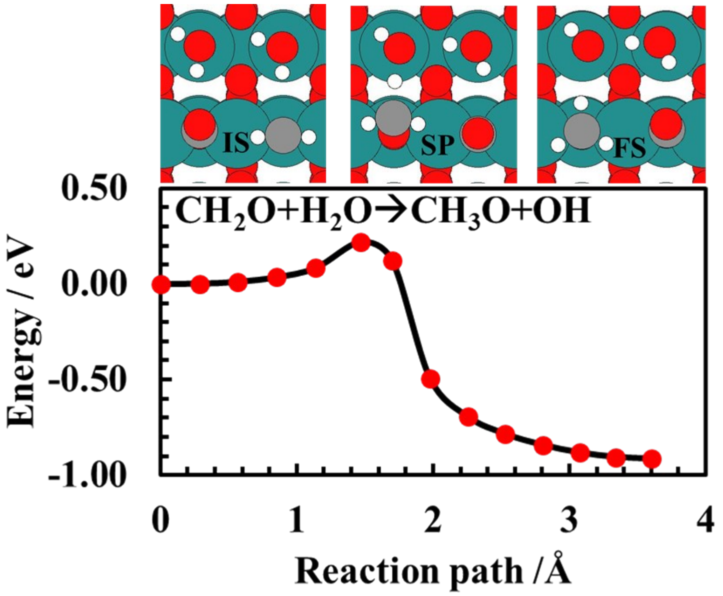

Figure S18. DFT calculated barrier for CH_2O protonation to CH_3O on $RuO_2(110)$ surface. (IS) initial state, (SP) saddle point and (FS) final state are shown on the top of the figure.

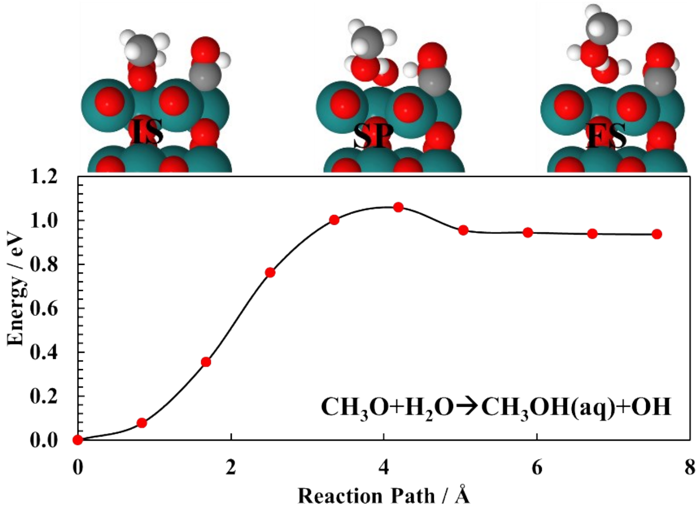

Figure S19. DFT calculated barrier for CH_3O protonation to $CH_3OH(aq)$ on $RuO_2(110)$ surface. (IS) initial state, (SP) saddle point and (FS) final state are shown on the top of the figure.

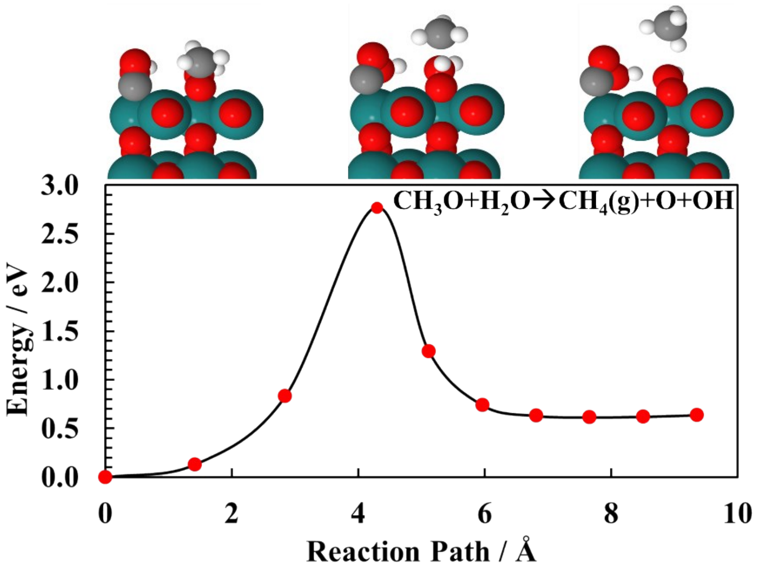

Figure S20. DFT calculated barrier for CH_3O protonation to CH_4(g) on RuO_2(110) surface. (IS) initial state, (SP) saddle point and (FS) final state are shown on the top of the figure.

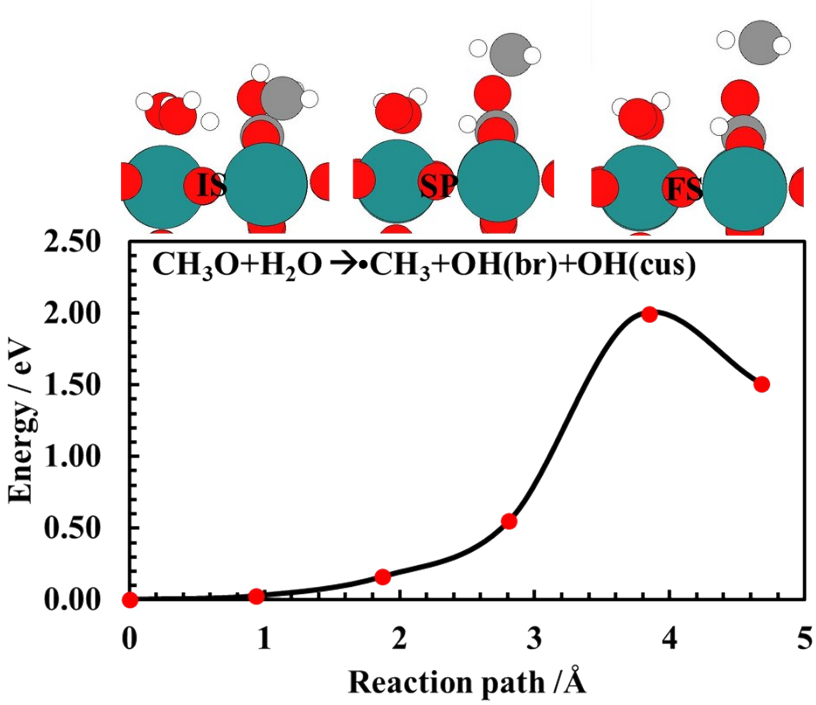

Figure S21. DFT calculated barrier for CH_3O protonation to $\bullet CH_3$+OH(br)+OH(cus) on RuO_2(110) surface. (IS) initial state, (SP) saddle point and (FS) final state are shown on the top of the figure.

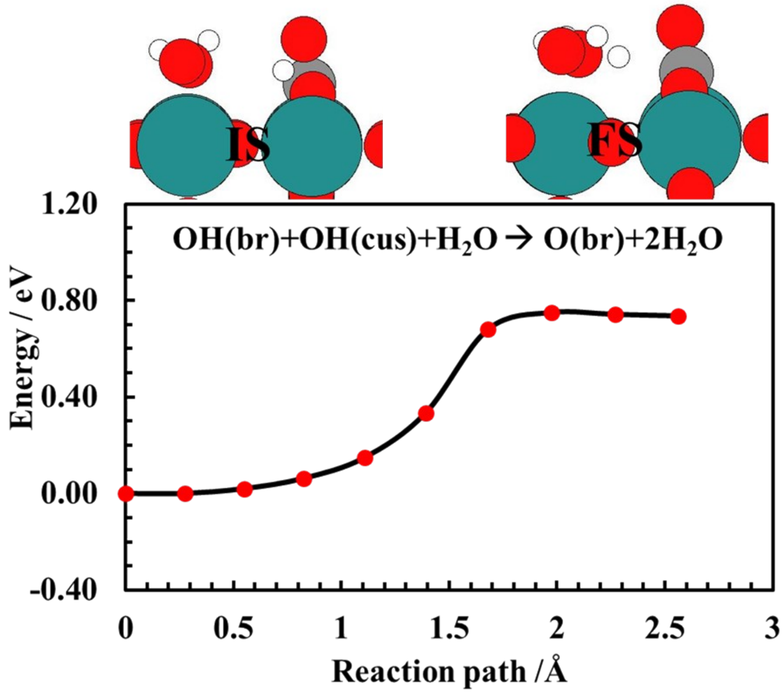

Figure S22. DFT calculated barrier for OH (br) reconfiguration to O(br) on RuO_2(110) surface. (IS) initial state, and (FS) final state are shown on the top of the figure.

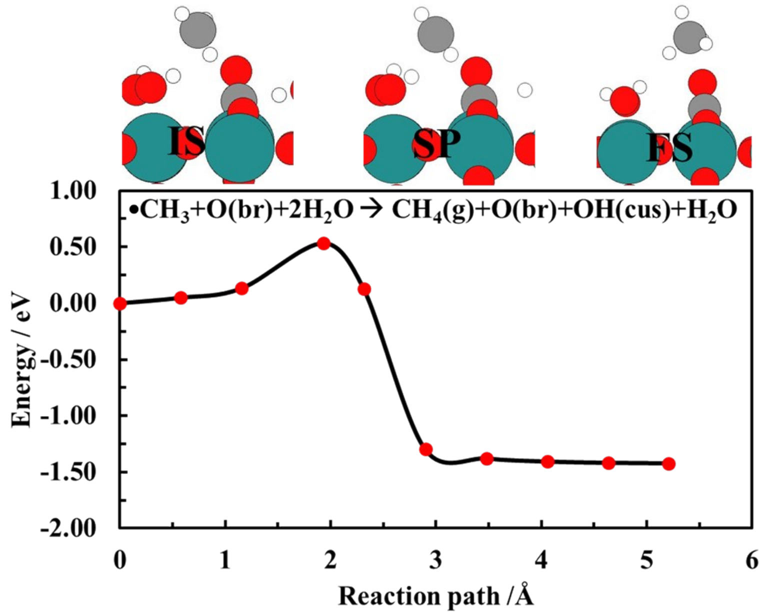

Figure S23. DFT calculated barrier for $\bullet CH_3$ protonation to CH_4(g) on RuO_2(110) surface. (IS) initial state, (SP) saddle point and (FS) final state are shown on the top of the figure.

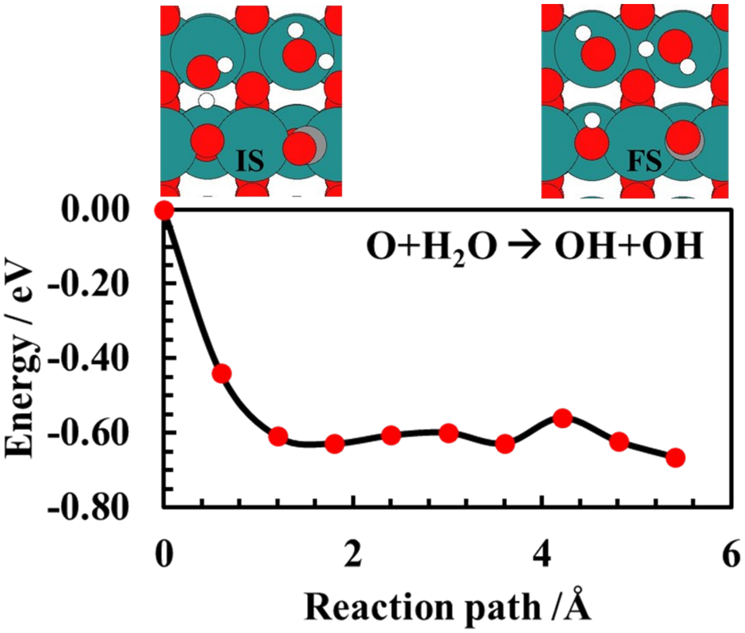

Figure S24. DFT calculations for O protonation to OH on RuO_2(110) surface. (IS) initial state and (FS) final state are shown on the top of the figure.

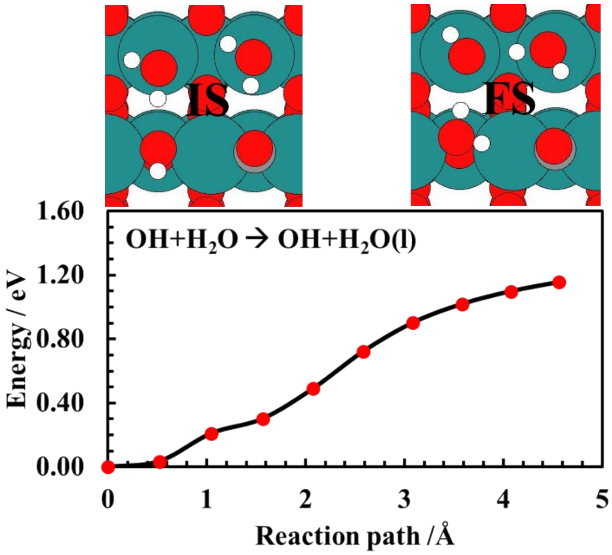

Figure S25. DFT calculations for OH protonation to H_2O(l) on RuO_2(110) surface. (IS) initial state and (FS) final state are shown on the top of the figure.

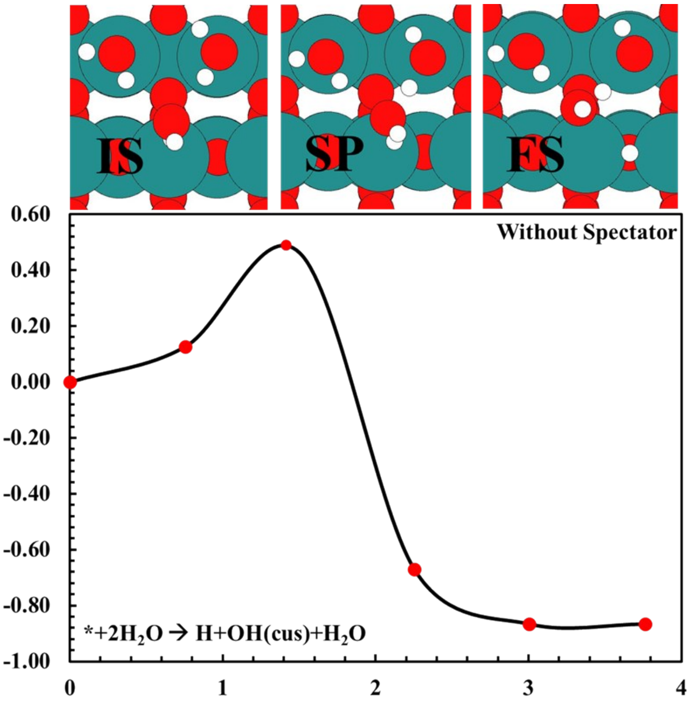

Figure S26. DFT calculations for transferring H to vacant bridge site on $RuO_2(110)$ surface. (IS) initial state, (SP) saddle point and (FS) final state are shown on the top of the figure.

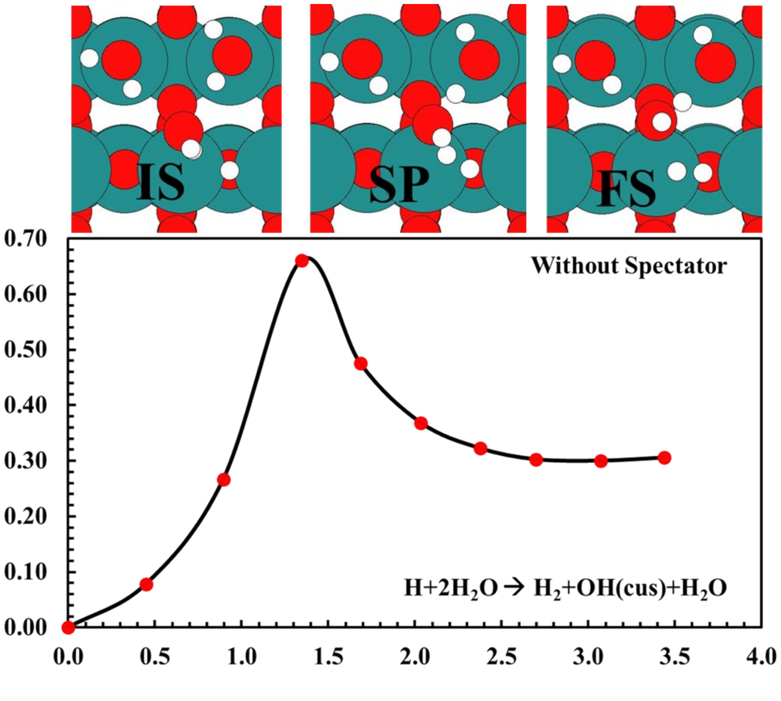

Figure S27. DFT calculations for H protonation to H_2 on $RuO_2(110)$ surface. (IS) initial state, (SP) saddle point and (FS) final state are shown on the top of the figure.

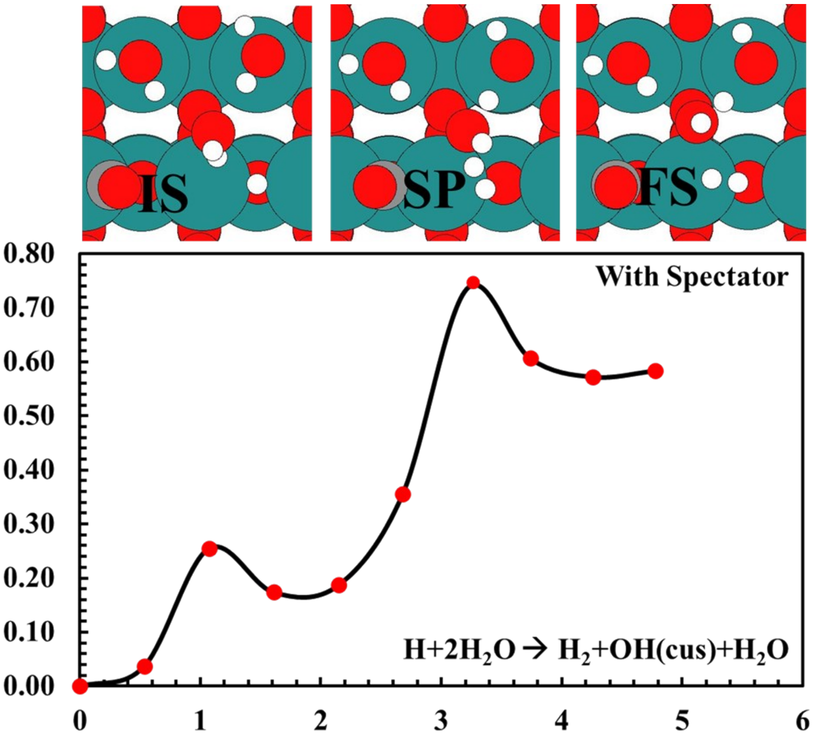

Figure S28. DFT calculations for H protonation to H_2 on RuO_2(110) surface where CO is spectator. (IS) initial state, (SP) saddle point and (FS) final state are shown on the top of the figure.

References

1 A. Bandi, *J. Electrochem. Soc., 1990, **137**, 2157.*

2 A. Bandi, *J. Electrochem. Soc., 1992, **139**, 1605.*

3 J. P. Popić, M. L. Avramov-Ivić and N. B. Vuković, *J. Electroanal. Chem*., 1997, **421**, 105–110.

4 N. Spataru, K. Tokuhiro, C. Terashima, T. N. Rao and A. Fujishima, *J. Appl. Electrochem*., 2003, **33**, 1205–1210.

5 Qu, J., Zhang, X., Wang, Y. & Xie, C. *Electrochim. Acta* **50**, 3576–3580 (2005).

6 S. Mezzavilla, Y. Katayama, R. Rao, J. Hwang, A. Regoutz, Y. Shao-Horn, I. Chorkendorff and I. E. L. Stephens, *J. Phys. Chem. C*, 2019, **123**, 17765–17773.
